# A novel machine learning-based algorithm for eQTL identification reveals complex pleiotropic effects in the MHC region

**DOI:** 10.1093/bib/bbag238

**Published:** 2026-05-19

**Authors:** Ronnie Y Li, Chang Su, Zhaohui S Qin

**Affiliations:** Neuroscience Graduate Program, Emory University, 1462 Clifton Road NE, Suite 314, Atlanta, GA 30322, United States; Department of Biostatistics and Bioinformatics, Emory University, 1518 Clifton Rd NE, Atlanta, GA 30322, United States; Department of Biostatistics and Bioinformatics, Emory University, 1518 Clifton Rd NE, Atlanta, GA 30322, United States

**Keywords:** expression quantitative trait loci (eQTLs), machine learning, gene expression, multivariate association, genome-wide association study (GWAS), genotype–phenotype association

## Abstract

Expression quantitative trait loci (eQTLs) are regulatory variants that affect the expression level of their target genes and have significant impact on disease biology. However, eQTL mapping has been done mostly in one tissue at a time, despite the known prevalence of correlations among tissues. Multivariate analyses incorporating multiple phenotypes are available, but they emphasize linear combinations of phenotypes. We present MTClass, a machine learning framework that attempts to classify an individual’s genotype based on a vector of multiphenotype expression levels of a given gene. We conduct simulation studies and multiple case studies using real and imputed data, and we demonstrate that MTClass detects more functionally relevant variants and genes compared to existing single-tissue approaches as well as multi-phenotype association tests. Our results suggest that the importance of expression regulation at the MHC region may have been underestimated, and they provide fresh biological insights into genetic variants that have pleiotropic effects, influencing gene expression in a complex manner.

## Introduction

Evaluating the consequences of genetic variation on the biological functioning on cells, tissues, and disease risks is an important undertaking in genetics. Widely adopted genome-wide association studies (GWASs) have revealed that most trait-associated variants lie in noncoding regions of the genome [1, 2], leading to the hypothesis that these variants affect regulatory functions instead of protein-coding ones. Kapoor and colleagues identified 210 common risk variants for QT interval variation; strikingly, all of them were noncoding [3]. Similar evidence has been found for other phenotypes like restless leg syndrome [4] and schizophrenia [5]. Indeed, such discoveries have spawned a new field in human genetics studying expression quantitative trait loci (eQTLs) [6].

An eQTL is a genetic variant that partially explains the variance in the expression level of a gene either proximally (in *cis*) or distally (in *trans*). Standard eQTL mapping involves a direct association test between the genotypes of a variant and a gene’s expression levels. Mapping and linkage studies have consistently shown that eQTLs have considerable biological significance [6]. GWAS-significant single nucleotide polymorphisms (SNPs) are more likely to colocalize with eQTLs [7]. Additionally, eQTLs are more likely to have regulatory implications such as altering transcription factor binding affinity [8]. They can also be organized into tissue-specific networks to inform disease risk and coherent biological processes [9].

Despite the significance of eQTLs, gene expression levels are tissue-specific, and most existing eQTL studies have focused on identifying eQTLs in one tissue at a time. However, somatic tissues are statistically and biologically correlated [10], raising the possibility that an eQTL can affect gene expression in more than one tissue. Nevertheless, current approaches to detect the pleiotropic interaction between one genotype and multiple phenotypes are relatively limited. Notable examples include MultiPhen [11], MANOVA [12], SCOPA/META-SCOPA [13], MASH [14], and others [15–18]. One significant limitation of these methods is that they only consider linear effects of genotype on phenotype, which is rather restrictive.

**Figure 1:**
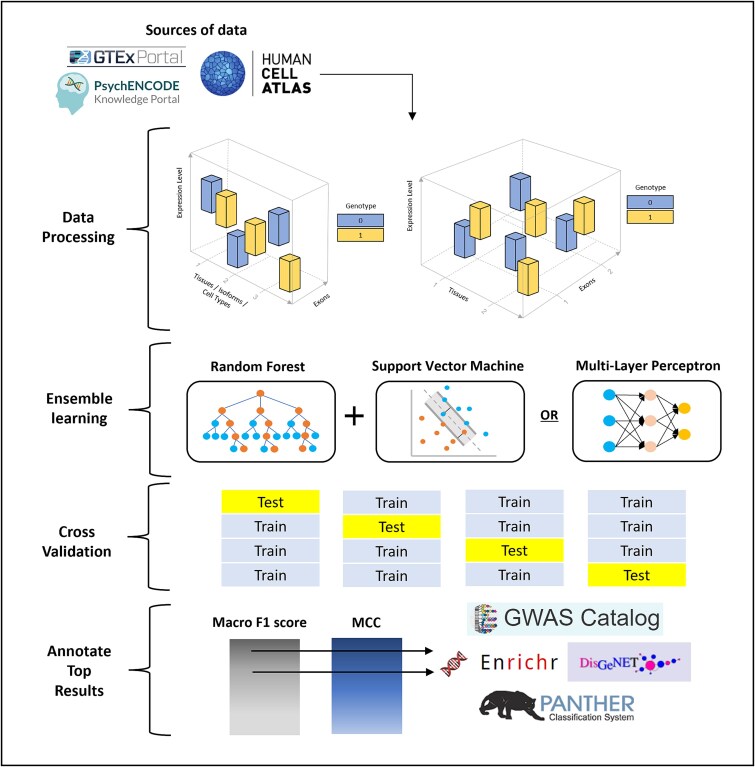
Schematic overview of MTClass algorithm. After processing the data from the GTEx Consortium, the PsychENCODE Consortium, and the Human Cell Atlas, MTClass uses an ensemble learning framework comprised of random forest and support vector machine, combined with four-fold cross-validation, to classify genotypes according to the vector of expression levels from a nearby gene. Variants are then sorted by macro F1 or MCC, and the top variants are annotated using various bioinformatic databases.

In this study, we present MTClass, a model-free approach adopting ensemble machine learning to classify a genotype based on multivariate gene expression levels from multiple sources (Fig. 1). Instead of calculating a *P*-value under the traditional statistical testing framework, we rank eGene–eQTL pairs based on classification performance. In doing so, we hypothesize that variants with superior classification performance are more likely to be multi-phenotype eQTLs. We apply MTClass to real datasets from the GTEx Consortium [19], the PsychENCODE Consortium [20–22], and the Human Cell Atlas [23], and we compare our method to state-of-the-art existing approaches, demonstrating that our top-identified variants and genes are likely to have more functional relevance. Finally, we illustrate interesting anecdotal examples of multi-phenotype eQTLs.

## Results

### Simulation studies

To evaluate the relative performance of our method in detecting nonlinear relationships, we conducted two numerical simulation studies with simulated gene expression matrix in which the ground truth (genotype class labels) is known. We found that while all methods achieve perfect results when the eQTL effect is linear, MTClass substantially outperforms competing methods given complex, nonlinear genotype–phenotype relationships that violate linear assumptions. Moreover, we have shown that despite its default binary genotype encoding scheme, MTClass is capable of reliably detecting additive and multiplicative eQTL effects, although it excels when the genetic effect is aligned with the encoding. Details of the simulations can be found in the Supplementary Materials.

### Multi-tissue study

We enumerate three cases with different subsets of donors and tissues to demonstrate the utility of our approach: the 9-tissue case, the brain tissue case, and the 48-tissue case. The 9-tissue case is carefully selected such that there is no missing data, while the brain tissue case and 48-tissue case contain a substantial amount of missing data. Table 1 lists the sample sizes, number of genes, variants, and features used for each study.

**Table 1:** Number of unique eGenes, variants, donors, and features used in all experiments. Features refer to tissues (multi-tissue study), exons (multi-exon study), both tissues and exons (2D study), isoforms (isoQTL study), or cell types (OneK1K study).

Experiment	Genes	Variants	Sample size	Number of features
Multi-tissue studies				
9-tissue	13 811	3 013 552	103	9
Brain tissue	13 812	3 017 214	317	13
48-tissue	13 813	3 018 097	838	48
Multi-exon studies				
Brain_Amygdala	5040	1 854 146	129	Varies by gene
Brain_Anterior_cingulate_cortex_BA24	5045	1 855 259	147	Varies by gene
Brain_Caudate_basal_ganglia	5049	1 859 142	194	Varies by gene
Brain_Cerebellar_Hemisphere	5044	1 842 548	175	Varies by gene
Brain_Cerebellum	5051	1 861 331	209	Varies by gene
Brain_Cortex	5050	1 853 926	205	Varies by gene
Brain_Frontal_Cortex_BA9	5050	1 860 706	175	Varies by gene
Brain_Hippocampus	5049	1 860 066	165	Varies by gene
Brain_Hypothalamus	5050	1 852 746	170	Varies by gene
Brain_Nucleus_accumbens_basal_ganglia	5052	1 849 364	202	Varies by gene
Brain_Putamen_basal_ganglia	5049	1 859 289	170	Varies by gene
Brain_Spinal_cord_cervical_c-1	5041	1 846 537	126	Varies by gene
Brain_Substantia_nigra	5037	1 848 812	114	Varies by gene
Other studies				
2D (multi-tissue/multi-exon)	10 227	3 857 513	103	Varies by gene
isoQTL (PsychENCODE)	5435	1 265 533	900	Varies by gene
OneK1K (scRNA-seq)	9237	1 251 974	982	7

#### 9-tissue case

**Figure 2:**
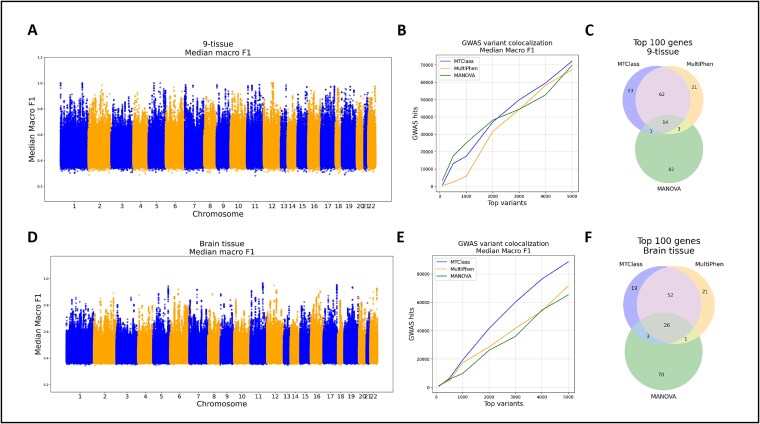
Functional assessment of top variants identified by MTClass compared to MultiPhen and MANOVA in the multi-tissue study. (A)–(C) refer to the 9-tissue case, and (D)–(F) refer to the brain tissue case. (A) Manhattan plot of variants according to their median macro F1 score. (B) GWAS variant colocalization analysis of top variants from each method. (C) Overlap of the top 100 genes detected by each method. (D) Manhattan plots of variants by median macro F1 score. (E) GWAS variant colocalization analysis of top variants. (F) Overlap of the top 100 genes detected by each method.

The 9-tissue case consisted of gene-level expression data from 9 tissue types across 103 donors. The nine tissues included subcutaneous adipose, tibial artery, lung, skeletal muscle, tibial nerve, skin (not sun-exposed, suprapubic), skin (sun-exposed, lower leg), thyroid, and whole blood. We chose these nine tissues to maximize the number of tissues while maintaining sufficient sample sizes without missing data. To ensure that our results were reproducible and consistent, we ran our classifier three times on the same dataset, each time with different random seeds, then aggregated the macro F1 scores [24] and Matthews correlation coefficient (MCC) [25] by calculating the median across the three iterations. Figure 2A illustrates the Manhattan plot for the 9-tissue case, using the median macro F1 score as the classification metric. Switching to median MCC to rank the top variants and genes triggered minimal change to the results, as the two metrics are highly correlated (Supplementary Fig. 1). A total of 54 eGene–eQTL pairs belonging to 8 genes achieved both perfect median F1 scores and median MCC in the three iterations of MTClass (Table 2). For comparison, we also ran MultiPhen and MANOVA on the same datasets. We noticed that there was a notable degree of overlap among the top 1000 and top 5000 variants selected by all three methods. Among the three top 1000 variants lists, 373 variants (37.3%) were common to all three methods, and among the top 5000 variant lists, there were 2913 common variants (58.3%, Supplementary Fig. 2). The top-performing eQTLs of the top 10 eGenes ranked by MCC in this study and in subsequent studies are summarized in Table 3.

**Table 2:** The 54 eGene–eQTL pairs that achieved perfect classification performance (median macro F1 score and median MCC) in three iterations of MTClass in the 9-tissue case. These eGenes are enriched for housekeeping processes involving DNA metabolism and processing, as well as cell division processes.

BTNL3	CLECL12A	DDX11	HORMAD1
rs72494581	rs111930678, rs113362278	chr12_31080603_GA_G_b38	chr1_150730558_CAGA_C_b38
	rs12230638, rs12231872	rs10843879, rs11051236	rs112718432, rs11803940
	rs199560361, rs200911123	rs2543261, rs2553134	rs11807450, rs1336899
	rs201541579, rs61913537	rs2553147, rs3881296	rs1336900, rs2184833
		rs3930906, rs4031323	rs3768005, rs6700022
		rs4031341, rs4031360	rs868751
		rs7133363, rs71440936	
		rs7309520	
**PDK1P5**	**PPIE**	**RPS26**	**ZNF300P1**
rs4781810	chr1_39739455_TAAAG_T_b38	rs1131017	rs12659118, rs76433514
rs62043986	rs1046988, rs11809515		
	rs12094291, rs2274949		
	rs3738673, rs41267029		
	rs56064244, rs58589165		
	rs6664570, rs6670841		
	rs7513045, rs7520588		
	rs7547787, rs7548120		
	rs9787249		

**Table 3:** The top-performing eQTLs from the top 10 eGenes by MCC in the 9-tissue case, the brain tissue case, and the 48-tissue case (multi-tissue study), the PsychENCODE isoQTL study, and the OneK1K scRNA-seq study.

Gene	Variant	F1 macro median	MCC median	Consequence
**9-tissue case**				
PPIE	rs41267029	1	1	intron
RPS26	rs1131017	1	1	downstream_gene
CLEC12A	rs199560361	1	1	intron
HORMAD1	chr1_150730558_CAGA_C_b38	1	1	Unknown
PKD1P5	rs4781810	1	1	intron
ZNF300P1	rs12659118	1	1	intron
DDX11	rs7309520	1	1	intron
BTNL3	rs72494581	1	1	intron
PSMD5-AS1	rs4837796	0.988	0.978	downstream_gene
LDHC	rs2119600325	0.987	0.976	downstream_gene
**Brain tissue case**				
LDHC	rs3740714	0.969	0.939	downstream_gene
KANSL1-AS1	chr17_46201644_GGAGGATCATTT_G_b38	0.952	0.905	Unknown
RPS26	rs1131017	0.949	0.898	downstream_gene
ERAP2	rs2927608	0.944	0.889	intron
CYP4F24P	rs919811	0.935	0.871	intron
FAM118A	rs2350630	0.926	0.856	3_prime_UTR
RNF39	rs11758516	0.919	0.851	5_prime_UTR
RP11-613E4.4	rs6972291	0.917	0.835	upstream_gene
DDX11	rs2005896	0.914	0.83	intron
RP11-109 L13.1	rs4936365	0.909	0.821	intron
**48-tissue case**				
RPS26	rs1131017	0.992	0.983	downstream_gene
PKD1P5	rs62043986	0.985	0.971	intron
NUPR2	rs763336281	0.986	0.971	intron
LDHC	rs11605386	0.985	0.97	downstream_gene
C17orf97	rs7503725	0.983	0.967	synonymous
KANSL1-AS1	rs2732672	0.982	0.965	intron
FAM118A	rs2142552	0.979	0.959	intron
ERAP2	rs2910686	0.978	0.957	intron
XRRA1	rs2119028	0.972	0.945	upstream_gene
PSMD5-AS1	chr9_120843433_CGAAGGCGTGAGTAATA_C_b38	0.97	0.94	Unknown
**2D study**				
GTF2IP12	rs71614440	1	1	upstream_gene
KANSL1	rs62060954	1	1	upstream_gene
CRYBB2P1	rs5996939	1	1	intron
FAM118A	rs2142552	1	1	intron
LERFS	chr9_62880578_G_A_b38	1	1	upstream_gene
PKD1P5	rs4781810	1	1	intron
PKD1P4	rs764068030	0.983	0.969	non_coding_transcript_exon
ANKRD36B	rs6744616	0.981	0.965	intron
SMG1P5	rs138082532	0.981	0.963	intron
SH3YL1	rs62114506	0.98	0.963	intron
**PsychENCODE isoQTL study**				
LDHC	rs77724297	0.951	0.903	intron
SH3YL1	rs62114501	0.942	0.885	intron
COA1	rs73108637	0.927	0.864	intron
BTN3A3	rs9461252	0.919	0.844	upstream_gene
CDH18	5_19868699_C_T_b37	0.918	0.837	intron
ARHGEF3	rs6787106	0.908	0.824	downstream_gene
SCAPER	rs11857015	0.893	0.789	intron
RPGRIP1L	rs7203525	0.89	0.783	intron
RNH1	rs11822000	0.879	0.763	intron
NME7	rs10800418	0.873	0.748	intron
**OneK1K scRNA-seq study**				
ABO	rs11244049	0.971	0.943	downstream_gene
LINC01954	rs7577665	0.963	0.927	intron
FAM118A	22_45739322_C_T_b37	0.958	0.916	downstream_gene
SCGB3A1	5_180007693_G_A_b37	0.954	0.909	downstream_gene
C20orf204	rs816933	0.934	0.869	intron
CFAP95	rs7029206	0.927	0.855	upstream_gene
CENPK	5_64858687_C_G_b37	0.902	0.804	upstream_gene
ENSG00000272501	rs6899874	0.9	0.803	upstream_gene
KRT1	rs2741159	0.899	0.798	intron
RPS26	CR1310395	0.874	0.749	downstream_gene

Next, we tested whether the top variants identified by MTClass were more functionally relevant compared to the top variants identified by MultiPhen and MANOVA. To do this, we downloaded the GWAS Catalog [26] and examined whether each method’s top variants were colocalized with known trait-associated SNPs from the catalog. We defined “GWAS hits” as the total number of unique trait-associated SNPs within 10 kilobases of a given variant’s genomic position. After selecting the same number of top variants from MultiPhen and MANOVA by *P*-value, we calculated the GWAS hits for the top 5000 variants selected by each method.

We demonstrated that the top variants selected by MTClass colocalized with more known GWAS-identified trait-associated SNPs than did those selected by MultiPhen, regardless of whether we sorted by median macro F1 score or median MCC (Fig. 2B). MTClass performed similarly to MANOVA overall in terms of GWAS hits. However, MTClass outperformed MANOVA in the latter half of the variants tested.

Among the top 100 genes, only 14 genes (14%) were common to all three methods, suggesting that each method detected distinct patterns in the data (Fig. 2C). We analyzed the functional potential of the eight eGenes containing perfectly classified eQTLs using PantherDB [27] and Enrichr [28], and we discovered that they were enriched for DNA metabolism and processing, as well as cell division processes (Supplementary Fig. 3).

#### Brain tissue case

There are 13 brain tissues profiled in the GTEx Consortium. The v8 version of the GTEx data contains 317 donors contributing at least one tissue type. The average percentage of missing data across all 13 tissues was 47% (between 33% and 64%, Supplementary Fig. 4). Expression levels from missing donor-tissue combinations were imputed for each gene using predictive mean matching with chained equations [29] (see Supplementary Materials). We implemented the imputation strategy five times and took the arithmetic mean.

We ran our classifier three times and computed the median macro F1 score and median MCC for each eGene–eQTL pair. Due to large amounts of missing data, the overall classification performance in the brain tissue case was poorer compared to that in the 9-tissue case, as evidenced by the Manhattan plots (Fig. 2D). Although no eGene–eQTL pairs achieved a median macro F1 score or MCC equal to 1.0, we still observed evidence of strong classification performance among the top variants.

Next, we tested whether the top variants and genes identified by MTClass were more functionally relevant compared to those identified by other methods. We found that the top variants from MTClass had substantially more colocalization with known trait-associated SNPs compared to those from MultiPhen and MANOVA (Fig. 2E). Among the top 100 eGenes detected by MTClass, MultiPhen, and MANOVA, 26 genes were common to all three methods (Fig. 2F).

#### 48-tissue case

The 48-tissue case was comprised of expression level vectors from 48 somatic tissues (including all 13 brain tissues) across 838 donors. The goal of this case study was to identify the most broad-acting eQTLs that exert effects across a very large number of tissues. We excluded tissues that had fewer than 100 donors available, reducing the total number of tissues from 54 to 48. Across the 48 tissues, the average missing data rate was 62%, ranging from 15% (skeletal muscle) to 85% (uterus and substantia nigra). As with the brain tissue case, we imputed missing transcripts per million (TPMs) for each gene using 5 iterations of predictive mean matching using chained equations, then taking the average of the predictions.

We repeated the GWAS variant colocalization analysis in the 48-tissue case and compared our results to MultiPhen and MANOVA. Although MTClass consistently outperformed MultiPhen in terms of neighboring GWAS hits, it only outperformed MANOVA in the latter portion of the top variants tested (top 2500–5000 variants; Supplementary Fig. 5).

### Multi-exon study in brain tissue

Alternative splicing is a major regulator of gene expression, particularly in the human brain [30, 31]. Studies have shown that aberrations in alternative splicing contribute to many disorders, including frontotemporal dementia [32], Duchenne muscular dystrophy [33], and cystic fibrosis [34]. By studying multi-exon eQTLs, we hope to identify variants that affect the expression of multiple exons within the same gene and thereby contribute to alternative splicing mechanisms or differential expression of transcript isoforms. Identifying these genetic variants can provide insight into both regulation of gene expression and alternative splicing, as exon-level expression might be more informative than gene-level expression [35, 36].

We adapted our multi-tissue approach so that the features in our model were expression levels from various *exons* of the same gene in a single tissue. To maximize the chance of novel discovery, we focused on exon-rich genes defined as genes containing at least 10 exons. Altogether, we processed about 5000 genes per tissue (the exact number of genes varied slightly by tissue and is listed in Table 1). We ran three iterations of MTClass in each of 13 brain tissues obtained from the GTEx Consortium. We compared our approach to MultiPhen and MANOVA. The top-performing eQTLs of the top 10 eGenes ranked by MCC from each tissue are summarized in Supplementary Data 1.

**Figure 3:**
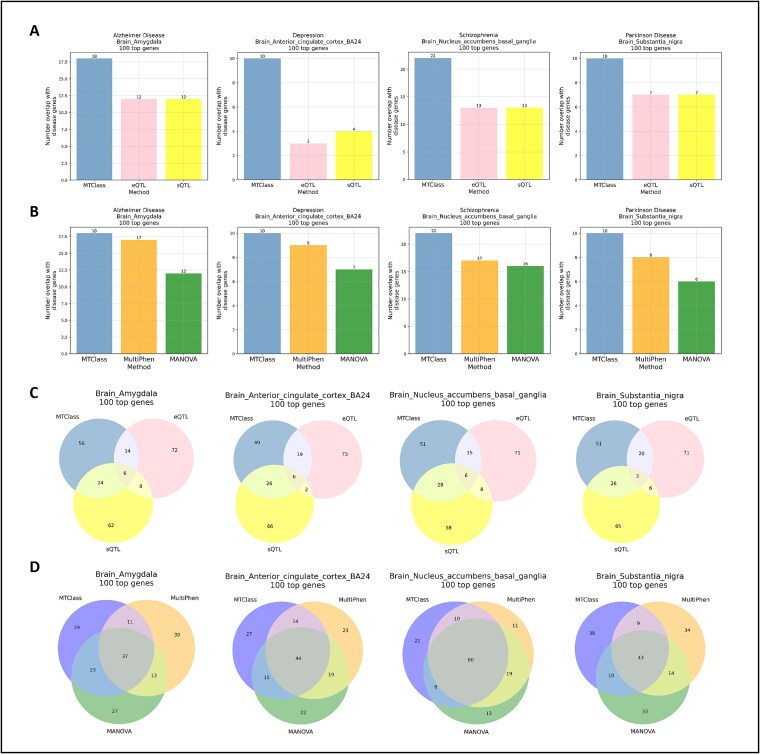
Functional assessment of top genes identified by MTClass compared to single-tissue studies and other multivariate methods in the multi-exon study in the brain. All overlap analyses were conducted using the DisGeNET database, version 3. (A) Overlap with known disease-associated genes among the top 100 genes compared to single-tissue eQTL and single-tissue sQTL methods. (B) Overlap with known disease-associated genes among the top 100 genes compared to MultiPhen and MANOVA. (C) Overlap among the top 100 genes detected by single-tissue methods. MTClass largely detects distinct top genes from single-tissue eQTL and sQTL methods. (D) Overlap among the top 100 genes detected by MTClass compared to MultiPhen and MANOVA.

To determine whether the top genes identified by MTClass in each brain tissue were more functionally relevant than those identified by MultiPhen or MANOVA, we focused on four neurological and psychiatric disorders that are known to substantially impact specific areas of the brain: Alzheimer’s disease (AD) and the amygdala [37, 38], depression and the anterior cingulate cortex [39, 40], schizophrenia and the nucleus accumbens [41, 42], and Parkinson’s disease (PD) and the substantia nigra [43]. Using version 3 of the DisGeNET database [44], a comprehensive knowledge base of disease-gene associations, we calculated the amount of overlap between the top genes from each method and known disease-related genes. We selected the top 100 eGenes from the MTClass, MultiPhen, and MANOVA results as well as the top 100 eGenes from the single-tissue eQTL and splicing QTL (sQTL) analyses. Throughout these four comparisons, we found that the top 100 eGenes detected by MTClass had the greatest overlap with disease-related genes in these four disease-related tissues, outperforming both single-tissue eQTL and sQTL approaches (Fig. 3A) as well as MultiPhen and MANOVA (Fig. 3B). We also discovered that the top genes detected by MTClass were largely distinct from those detected by other methods, although MTClass shared more top genes with MultiPhen and MANOVA than with single-tissue eQTL and sQTL methods (Fig. 3C and D).

### Multi-tissue and multi-exon 2D study

Given the highly correlated nature of gene expression among exons and tissues, a third consideration is that one eQTL can have far-reaching effects across both exons and tissues. We tested this possibility by using a two-dimensional feature matrix of multiple exons across nine tissues for each gene as input to our model. We used the same tissues as in the 9-tissue case to avoid imputing missing data. The model we chose for this study was an MLP, or fully connected neural network, to facilitate the capture of any latent structure among the expression levels.

We also tried the same data (as a flattened vector) on MultiPhen and MANOVA; however, these two methods encountered multiple numerical issues due to the high dimensionality and abnormal correlation pattern of the data. Thus, we decided not to pursue a performance comparison on this dataset. We ran only one iteration of MTClass on this dataset due to the relatively higher computational burden. We provide interesting anecdotal examples of our findings in the Supplementary Materials.

### PsychENCODE isoQTL study

In vertebrate eukaryotic species, alternative splicing generates an enormous amount of mRNA diversity by facilitating the formation of multiple mRNA isoforms. Understanding the contribution of genetic variation to the abundance of various mRNA isoforms will provide a more comprehensive understanding of alternative splicing and neurological illness.

Because of the relatively small sample sizes available in the studies from the GTEx consortium, we turned to an independent dataset from the PsychENCODE Consortium [20, 22], specifically the subset of data from the CommonMind Consortium (CMC) [21]. Here, each feature was the expression level from each ‘isoform’ of a gene, obtained from post-mortem adult prefrontal cortex of the brain. We restricted our analyses to genes that had at least 10 isoforms to ensure there were enough features for our algorithm to consider. There was a total of 900 donors in this study. We term the top-scoring variants “isoQTLs” and their corresponding genes “isoGenes” because the variants are associated with the expression of multiple isoforms of a gene.

We ran MTClass three times on the same dataset with different random seeds. The hyperparameters were kept the same. MultiPhen and MANOVA were also run as a comparison. Subsequently, we conducted GWAS variant colocalization analysis on the top 5000 variants for each method. We found no marked differences in GWAS variant colocalization among the top variants detected by the three methods. However, we found that MTClass identified more isoGenes associated with anxiety disorder and bipolar disorder, two diseases that significantly affect prefrontal cortex function [45–48] (Supplementary Fig. 6). Moreover, we observed that for other prefrontal cortex-related disorders such as schizophrenia, autism spectrum disorder, attention-deficit hyperactivity disorder, and obsessive-compulsive disorder, MTClass always achieved as much, or even greater, disease-gene overlap as MultiPhen and MANOVA (Supplementary Fig. 6). Altogether, these results suggest that MTClass excels at recapitulating functionally important genes in the prefrontal cortex.

### OneK1K scRNA-seq study

Most eQTL studies have been conducted on bulk RNA-sequencing data, in which the data represent an average expression of a gene across all cells in a tissue [49]. The advent of single-cell RNA-sequencing (scRNA-seq) has revolutionized transcriptomics by allowing the quantification of cell-type-specific gene expression and the identification of cell type heterogeneity [50–53]. Cell-type-specific eQTLs have been identified in scRNA-seq studies as well, although this focuses on a single cell type at a time [23, 54]. It is likely that some eQTLs can have effects on more than one cell type. Thus, we extended our study to identify eQTLs that affect gene expression across multiple cell types.

**Figure 4:**
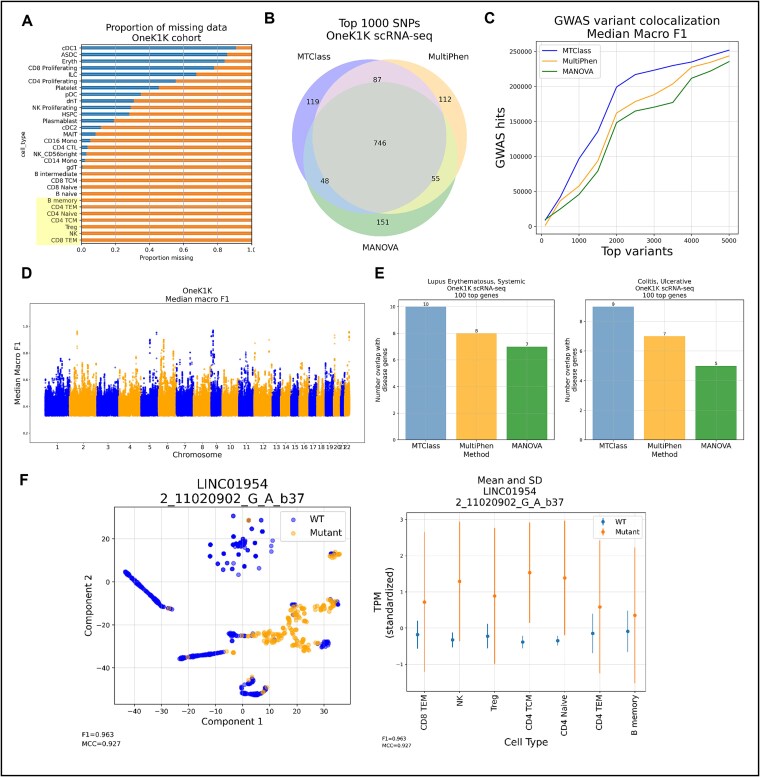
MTClass identifies eQTLs across multiple cell types in the OneK1K scRNA-seq cohort. (A) Visualization of missing data across different cell types in the dataset. The highlighted box indicates the seven cell types that were used as features for multi-cell-type eQTL identification. (B) There is a substantial amount of overlap in the top 1000 variants detected by each multivariate association method. (C) MTClass identifies variants that colocalize with more known trait-associated SNPs from the GWAS Catalog compared to MultiPhen and MANOVA. (D) Manhattan plot of MTClass results across three iterations by median macro F1 score. (E) The top MTClass genes overlap with more genes associated with various autoimmune diseases such as systemic lupus erythematosus and ulcerative colitis compared to the top genes from MultiPhen and MANOVA. (F) LINC01954-2_11020902_G_A_b37 (rs7577665) is an eQTL-eGene pair among MTClass’ top results that is not a known eQTL in whole blood from the GTEx Consortium data.

The OneK1K cohort is a notable example of the power of scRNA-seq, sequencing over a million cells from peripheral blood mononuclear cells (PBMCs) across nearly 1000 individuals [23]. We obtained this dataset from CELLxGENE, and we chose the seven major cell types that contained at least one cell for all 982 donors, resulting in no missing cell-type/donor combinations (Fig. 4A).

We ran our MTClass classifier on the OneK1K dataset, using expression measures from the seven major cell types as features for each gene, and we compared our approach to MultiPhen and MANOVA. We observed that there was a substantial amount of overlap among the top 100 genes detected by each of the three multivariate association methods (Fig. 4B), which could be due to the highly correlated nature among the seven selected cell types in the OneK1K cohort.

GWAS variant colocalization analysis revealed that the top variants identified by MTClass colocalized with more known signals in the GWAS Catalog compared to those identified by MultiPhen and MANOVA (Fig. 4C). Intriguingly, most of these signals originated from chromosome 6, on which many HLA genes reside (Supplementary Fig. 7). Figure 4D depicts the Manhattan plots for MTClass based on median macro F1 score and median MCC, highlighting the strong signals from chromosome 6. This evidence suggests that immune-related traits comprise most of the identified signals, which is biologically plausible because most of the seven cell types included in our study have important functions in immune signaling and antigen processing. Using the DisGeNET database, we found that the top eGenes selected by MTClass overlapped with more genes associated with various autoimmune disorders such as systemic lupus erythematosus and ulcerative colitis (Fig. 4E).

Among the top 10 MTClass results listed in Table 3, LINC01954-2_11020902_G_A_b37 (rs7577665) is an eGene–eQTL pair that is not a known pair in whole blood on the GTEx Portal. According to the GTEx Consortium, this pair is only statistically significant in the spleen. MTClass was able to classify this pair with high precision (macro F1 score = 0.963, MCC = 0.927). Indeed, dimensionality reduction using t-SNE shows that the genotypes are clearly separable. Across the seven cell types, the mutant genotype generally exhibits higher mean expression levels as well as greater variation in expression compared to the wild-type genotype (Fig. 4F).

### Epigenomic validation of top eQTLs

**Figure 5:**
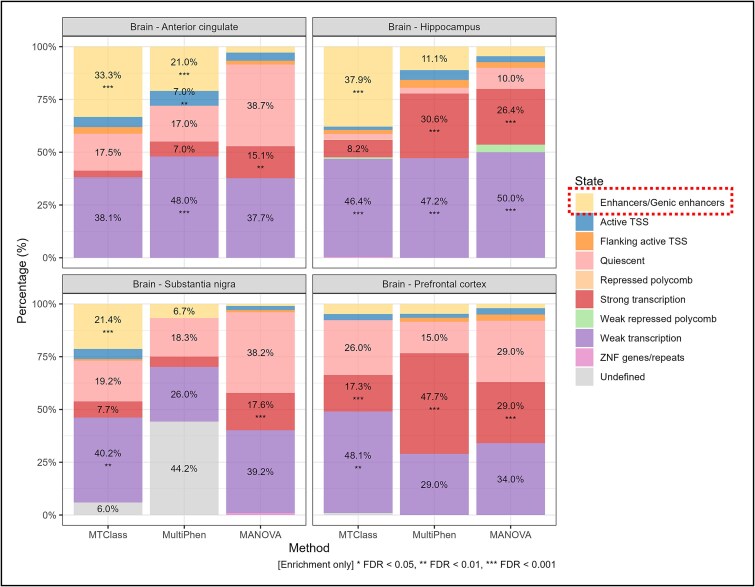
Epigenomic validation of the top 100 eQTLs in multi-exon study using the ChromHMM model from Roadmap Epigenomics. Chromatin state distributions for the top 100 eQTLs identified by MTClass, MultiPhen, and MANOVA across four brain tissues. The dotted box in the legend highlights the significant enhancer/genic enhancer enrichment in MTClass results for three of four tissues compared to minimal enrichment in MultiPhen and MANOVA. Asterisks denote enrichment significance relative to genome-wide proportions (Fisher’s exact test). ^*^indicates FDR < 0.05, ^**^indicates FDR < 0.01, ^***^indicates FDR < 0.001. MTClass-identified eQTLs preferentially localize to active regulatory regions, demonstrating superior prioritization of functionally relevant variants.

To assess functional relevance, we analyzed chromatin state enrichment using Roadmap Epigenomics’ [55] ChromHMM [56] annotations for individual brain tissues. Among the top 100 eQTLs per method, MTClass showed significant enhancer enrichment in 3/4 tissues versus 1/4 for MultiPhen and 0/4 for MANOVA. In substantia nigra, 21.4% of MTClass eQTLs overlapped enhancers (FDR < 0.001) compared to 6.7% (MultiPhen) and <5% (MANOVA). Similar patterns emerged in anterior cingulate (33.3% versus 21.0% versus <5%) and hippocampus (37.9% versus 11.1% versus <5%) (Fig. 5). MTClass variants concentrated in functionally active regulatory regions, providing orthogonal epigenomic evidence that our framework prioritizes regulatory-relevant eQTLs more effectively than linear methods.

### Anecdotal examples

One eQTL that was detected robustly by MTClass in the OneK1K scRNA-seq study was ABO-9_136123092_T_C_b37 (rs11244049), which is a downstream gene variant in the ABO gene on chromosome 9. This SNP achieved a median macro F1 score of 0.971 and a median MCC of 0.943. t-SNE dimensionality reduction analysis was able to segregate the genotypes quite well. This SNP seems to have a pronounced effect on ABO gene expression, with the mutant genotype having markedly higher mean expression and a greater variance compared to the wild-type genotype (Supplementary Fig. 8). Unsurprisingly, many of the top variants identified by MTClass modulate the ABO gene, which is responsible for human blood typing [57]. This result makes intuitive sense given that the data are from human peripheral blood. Further anecdotal examples from other studies are available in the Supplementary Materials.

### Comprehensive performance comparison

Several other statistical methods have been developed to perform multivariate genetic association. In this study, we consistently compared the performance of MTClass to MultiPhen [11] and MANOVA [12] to alleviate the computational burden of running all methods on all datasets. However, for the brain tissue case, we compared MTClass to a diverse array of methods to illustrate the superior performance of our approach. Notably, we utilized canonical correlation analysis (CCA, as implemented in mv-PLINK [58]), multivariate logistic regression (an adaptation of SCOPA/META-SCOPA [13]), and the Cauchy combination test [59] to combine *P*-values from single-tissue eQTL association studies. Specifically, we used GWAS variant colocalization analysis to evaluate the potential of each method at detecting functionally relevant eQTLs. We observed that MTClass outperformed all other methods on this metric in the brain tissue case (Supplementary Fig. 9), demonstrating that MTClass excels at detecting genetic variants with high functional importance.

### Enrichment of MHC variants

We observed that a large proportion of MTClass’s top eQTLs originated from chromosome 6, so we hypothesized whether signals in the MHC region could be driving the results. To ensure our results would not be driven by linkage disequilibrium (LD), we pruned variants in high LD (*R*^2^ > 0.50). After pruning, we still observed that MTClass demonstrated consistent enrichment for MHC variants across multiple studies. In the 9-tissue case, differential eQTLs (MTClass macro F1 ≥ 90th percentile, whereas MultiPhen/MANOVA *P*-values ≥10th percentile) showed 0.458% HLA variants versus 0.233% genome-wide (OR = 1.971, *P* = 1.545 × 10^−18^, Fisher’s exact test). Similar enrichment emerged in the brain tissue case (0.305% versus 0.230%, OR = 1.326, *P* = 0.00623) and most strikingly in OneK1K, where 4.300% of the top 1000 MTClass eQTLs were HLA variants versus 0.352% expected (OR = 12.729, *P* = 3.497 × 10^−31^). This pattern suggests MHC variants exhibit complex, nonlinear regulatory effects across tissues that linear methods inadequately capture, consistent with known pleiotropic effects and tissue-specific regulation in this genomically complex region.

## Discussion

Understanding how genetic variants influence transcription regulation is highly important. Given that the transcription process is tightly regulated, it is of little surprise to find that eQTLs can affect gene expression across multiple entities. In this study, we proposed to use machine learning algorithms to classify donor genotypes based on the vector of expression levels from multiple sources (e.g. tissues, exons, isoforms, and cell types). We compared our approach to other avant-garde linear approaches and showed that our method often detects eQTLs and eGenes with greater functional impact than other methods. In this way, we introduce a novel means of conducting multivariate association analyses, which could motivate the development of future multivariate trait association methods.

Three key findings underscore MTClass’s ability to capture complex regulatory architecture. First, our simulation studies demonstrated that when the difference in expression between genotypes is only in variance, linear methods such as MultiPhen and MANOVA fail to recover any of the top eQTL signals, while MTClass largely retains its performance. Second, we observed consistent enrichment of MHC variants among differentially detected eQTLs across multiple independent studies. This enrichment emerged organically from our analyses and aligns with established understanding that the MHC region harbors extensive pleiotropic effects, with highly pleiotropic variants showing strong correlation with tissue-shared eQTLs [60]. The MHC’s extreme LD, high polymorphism, and complex haplotype structures challenge traditional analytical approaches [61], yet MHC genes consistently appear in eQTL network communities across diverse tissues [9], suggesting coordinated regulatory effects that may require nonlinear modeling to fully capture. Lastly, chromatin state enrichment analysis using Roadmap Epigenomics revealed that MTClass-identified eQTLs in brain tissues show substantially greater overlap with active enhancer states compared to MultiPhen and MANOVA. This orthogonal epigenomic validation demonstrates that our classification framework prioritizes variants with *bona fide* regulatory potential, complementing our GWAS colocalization findings and providing biological validation that MTClass effectively identifies functionally relevant regulatory variants missed by linear methods.

Compared to existing methods, MTClass has the following advantages. First, it provides a generalized framework to conduct association studies without requiring *a priori* specification of the alternative hypothesis. The statistical literature offers numerous specialized tests for a wide range of distributional differences (variance tests (Levene’s [62], Bartlett’s [63], Fligner-Killeen [64], quadratic components [65]), mean-shift tests (MANOVA, MultiPhen), and others), each powerful for its targeted scenario. MTClass instead provides a classification-based, distribution-agnostic approach that does not impose a specific parametric form on the genotype-expression relationship and that detects variants with strong associations regardless of whether they manifest through mean differences, variance changes, or complex nonlinear patterns. Single-tissue eQTL mapping is a special instance of our framework with only one feature, and even in multivariate cases, MTClass effectively classifies variants affecting expression in only one phenotype. Second, our results demonstrated that MTClass identifies more functionally impactful eGenes and eQTLs than competing methods which are linear model-based, suggesting that classification-based methods are better at identifying non-linear patterns of separation. Lastly, MTClass is more flexible as it accepts a greater range of values as input, including features with zero expression in all donors. MultiPhen and MANOVA will both report numerical errors in this scenario. Therefore, we believe that MTClass is a more reliable and flexible approach compared to linear multivariate methods such as MultiPhen and MANOVA.

One limitation of this study was the relatively low sample size in the 9-tissue case and in the 2D exon-tissue case, where expression levels and genotypes were available for only 103 donors. This impacts machine learning methods more severely than parametric approaches since they make no distributional assumptions and require larger samples to detect patterns accurately. For this reason, by default, MTClass implements a binary classification scheme (collapsing heterozygous and homozygous alternate genotypes to ensure sufficient training samples per class given the limited sample size), which is consistent with the assumption of the dominant genetic model. Under other genetic models, such as additive and multiplicative models, although one of the two classes will consist of expression levels with different distributions, if a detectable genetic effect exists between the distributions, there will be sufficient differences in the expression levels between the two classes to enable binary classification models to identify such eQTLs. Future extensions of MTClass could include multi-class classification or regression to model more complex genetic effects, provided that sufficient sample sizes are available.

We utilized independent datasets of human prefrontal cortex from the PsychENCODE Consortium and the OneK1K single-cell cohort to alleviate this issue and validate our GTEx results. Indeed, we showed that in the PsychENCODE isoQTL study, MTClass detected genes that overlapped with more prefrontal cortex-related disorders. Moreover, we provided evidence that in the OneK1K cohort, GWAS variant colocalization analysis favored MTClass over both MultiPhen and MANOVA. Another limitation is the increased computational burden of MTClass. Using 8 CPU cores and 64 GB of memory, MTClass requires ~20 h to test 1 million gene–variant pairs, whereas MultiPhen and MANOVA complete the same analysis in ~3 h. However, MTClass provides key advantages over linear models, namely the lack of assumptions about linear relationships between genotype and expression and the robustness to multicollinearity. For these reasons, we believe that the increased computational time of MTClass is a worthwhile tradeoff. While MTClass primarily ranks variants by classification performance, permutation testing can generate *P*-values and effect sizes when integration with downstream frameworks like Mendelian randomization or transcriptome-wide association studies is needed. This serves as a bridge to existing statistical workflows rather than replacing the core classification-based ranking approach.

So far in genetics, all association tests are conducted under the hypothesis testing framework. Despite overwhelming success, this strategy does not work well for multivariate traits. In a recent study, Yu *et al.* successfully applied classification-based GWAS to full brain magnetic resonance (MR) images [66]. In this study, we showed that a similar strategy can be applied to quantitative traits such as eQTLs. To the best of our knowledge, this is the first time that a classification-based approach has been utilized to identify eQTLs. Even with basic machine learning approaches, MTClass demonstrates clear advantages over existing linear methods in detecting functionally relevant eQTLs. Future work could explore more sophisticated algorithms, but the classification framework itself, rather than the specific algorithm, is the key innovation. Future work should also aim to remove confounding effects from age, sex, and genotyping principal components and to account for variants in high LD while preserving superior performance.

Overall, identifying multi-phenotype eQTLs has the potential to elucidate biologically meaningful effects of genotype on gene expression and to provide insights into pleiotropic interactions. We are certainly not the first to propose that quantitative trait loci could affect disparate phenotypes such as gene expression, alternative splicing, and even polyadenylation [67]. We believe our results provide an exciting new avenue for conducting multivariate association analyses using machine learning.

## Methods

### GTEx data

A significant obstacle in mapping eQTLs in multiple tissues is limited tissue availability. The Genotype-Tissue Expression (GTEx) Consortium has exerted remarkable effort in overcoming this challenge by collecting many tissues from the same donors and performing rigorous quality control [19]. We used publicly available multi-tissue gene expression data in TPM obtained from the GTEx Consortium, version 8, consisting of 948 donors across 54 tissues, although the exact number of donors per tissue varied. We also utilized exon-level expression data across 13 brain tissues. The genotype data of the GTEx donors were accessed through dbGaP [68].

### Selection of genes and variants

To prioritize genes with diverse expression levels, we calculated the expression variance for all genes across all 17 382 tissue samples and excluded genes that had expression levels of zero in more than half of these samples. We then selected the top 50% of genes (*N* = 13 810) in terms of variance.

For each of the selected variable genes, we used PLINK to extract its candidate *cis*-eQTLs within 10 kb of the gene’s coding region and only retained variants that have a minor allele frequency of at least 0.05. In accordance with a dominant genetic model, the homozygous reference genotype was denoted as 0, while the heterozygous and homozygous mutant genotypes were denoted as 1. We combined genotypes that contained at least one mutant allele to ensure that there were enough samples with the least common genotype category for the machine learning algorithm to learn from the data. We removed variants and donors with a genotyping rate <90%, and we restricted our analysis only to autosomes to avoid potential issues with X-chromosome haploinsufficiency.

### Model selection and evaluation

Figure 1 depicts a schematic overview of the MTClass algorithm. The exact classifier we used depended on the input data. For single-dimensional vector inputs, we used the ensemble RF and SVM. For 2-dimensional inputs, we utilized the multi-layer perceptron to better handle the complexity of the feature space. We employed a soft-voting approach, where the predicted class for each sample was the argmax of the averaged probabilities of each base classifier. Details of the classifier intuition and architecture can be found in the Supplementary Materials.

In the multi-exon/multi-tissue 2D study, because our feature matrix was two-dimensional, we used an MLP with one hidden layer instead of an ensemble algorithm. This ensured that our network would be capable of detecting any latent structure among the features. We used *mn/2*, rounded up to the nearest integer, as the size of the single hidden layer, where *m* is the number of exons and *n* is the number of tissues.

We combined both the ensemble and MLP approaches with four-fold stratified cross-validation to mitigate model overfitting. We excluded variants that had fewer than four samples in the less common class so that each fold would have at least one sample with the less common class. Finally, we standardized each feature using the *z*-score method immediately prior to fitting our classifier. This procedure guaranteed that all the features would be on the same scale, which is critical for preventing bias in the machine learning model.

To evaluate classification performance, we used the macro F1 score and MCC. The F1 score is the harmonic mean between precision and recall, and the macro F1 score is the arithmetic mean of the per-class F1 scores. The MCC provides a balanced score between 0 and 1 that reflects the quality of predictions, considering all four values in the confusion matrix (see Supplementary Materials). Both metrics are robust to class imbalance, which was essential to our choice, as some eGene–eQTL pairs contained few samples of the less common class.

### Comparison with other methods

We compared our machine learning method with two state-of-the-art multivariate association methods, MultiPhen [11] and MANOVA [12]. Both methods output a single nominal *P*-value such that the ranking of the SNPs is possible for the purposes of performance evaluation. For this reason, we did not include methods such as MASH [14]. In the brain tissue case, we also compared the performance of MTClass to that of three additional methods: CCA, reverse logistic regression, and the Cauchy combination test. Additional information about these methods can be found in the Supplementary Materials.

Because MultiPhen and MANOVA sometimes reported numerical errors for certain eGene–eQTL pairs, we excluded features with expression levels of zero in all donors, allowing MultiPhen and MANOVA to conduct the association for a greater number of eGene–eQTL pairs. Lastly, we ensured all methods were tested on the same gene–variant pairs, and we excluded pairs for which any competing method reported a numerical error.

### GWAS variant colocalization analysis

GWAS variant colocalization analysis was done to assess the relative functional importance of the top variants identified by each method. We downloaded the entire GWAS Catalog [26] from the NHGRI-EBI website. Entries with duplicate or missing genomic positions were removed.

Because the GWAS Catalog uses the hg38 reference genome, we utilized the UCSC liftOver tool to convert the coordinates from hg38 to hg19 to be compatible with the genotype data from the PsychENCODE isoQTL and OneK1K scRNA-seq studies. In doing so, 138 coordinates could not be converted and were excluded from the analysis. The total number of unique entries left in the GWAS Catalog that contained both hg38 and hg19 genomic coordinates was 536 898.

We sorted the MTClass results by classification metric (macro F1 score or MCC) in descending order, and we sorted the results from all other methods by *P*-value in ascending order. Then, we selected the top *N* variants from the sorted results, and for each top variant, we computed the number of unique genomic positions in the GWAS Catalog that were within 10 kilobases of the variant’s genomic locus. We termed this number the “GWAS hits.”

When selecting the top *N* variants, it is possible that we are unable to select exactly *N* top variants due to ties in *P*-value or classification performance. To account for this, for each result, suppose the fewest number of variants that produce at least N top variants is *M*. Therefore, *M* ≥ *N*. We next calculated the number of GWAS hits for these M variants, then adjusted this count by *N*/*M*.

### Disease gene overlap analysis

We first downloaded disease-gene associations for various neurological diseases from version 3.0 of the DisGeNET (https://www.disgenet.com) database [44]. For each study, we filtered out disease genes that were not in the background gene list. Next, we selected the top 100 unique eGenes from the MTClass results after sorting by median macro F1 score in descending order. We first compared the MTClass result to single-tissue results by downloading the single-tissue eQTL and sQTL datasets from the GTEx Consortium. We sorted their eGenes by nominal *P*-value in ascending order, then selected the top 100 unique eGenes. We did the same with MultiPhen and MANOVA after sorting by nominal *P*-value in ascending order. Finally, we computed the intersection of the result sets with the filtered disease gene list to obtain the overlap.

### OneK1K scRNA-seq data processing

This study aimed to identify eQTLs across multiple cell types from single-cell RNA-sequencing data. We used PBMCs sequenced in the OneK1K cohort due to the large sample size. Raw scRNA-seq data were downloaded from CZ CELLxGENE Discover. The data were processed using the Seurat v4 package in R. First, the raw Unique Molecular Identifier (UMI) counts were log-normalized using default parameters. We retained the 3000 genes with the highest variance. We kept the 7 cell types with all 982 donors available, resulting in no missing data. These 7 cell types constituted ~77% of all predicted cells in the dataset, including the 4 most abundant cell types. Specifically, the chosen cell types were B memory, CD4 TEM, CD4 Naïve, CD4 TCM, T regulatory, natural killer (NK), and CD8 TEM. For each gene, the sum of the normalized expression levels of all cells (pseudobulk) belonging to a given cell type was taken as the expression measurement for that cell type. The features (cell types) were finally *z*-standardized before running the MTClass algorithm.

The genotype data for the OneK1K cohort were obtained via personal communication. We followed the same procedure to extract *cis*-eQTLs of interest as in the previous experiments. We compared our approach to MultiPhen and MANOVA. All methods were run on binarized genotypes in accordance with a dominant model.

### Epigenomic validation of top eQTLs

To assess functional relevance of identified eQTLs, we obtained chromatin state annotations from the Roadmap Epigenomics’ [55] ChromHMM [56] models for four brain tissues matching those in our multi-exon study (anterior cingulate cortex, hippocampus, substantia nigra, and prefrontal cortex). We focused on the multi-exon results because these analyses were conducted within single tissues, allowing direct comparison with tissue-matched chromatin states. We also analyzed six cell types from the OneK1K study for which Roadmap data were available. For each method (MTClass, MultiPhen, MANOVA), we extracted the top 100 eQTLs and determined the proportion overlapping each chromatin state. Enrichment significance was computed using Fisher’s exact test, comparing observed overlap proportions to genome-wide background proportions calculated from the complete set of tested eQTLs for that study. FDR correction was applied per tissue to account for multiple testing across chromatin states.

**Key Points**

- MTClass is a machine learning-based approach that classifies genotypes based on multi-phenotype expression data, providing a novel method for identifying eQTLs.
- MTClass demonstrates improved performance relative to traditional linear methods like MultiPhen and MANOVA in detecting eQTLs with greater functional impact and in capturing complex genotype–phenotype relationships.
- MTClass identified immune-related variants in the HLA region, suggesting that existing approaches may have underestimated the complexity of these variants’ effects across tissues.
- MTClass is more flexible and reliable than linear multivariate methods, handling multicollinearity, zero-expressed features, and various input values with greater ease.

## Supplementary Material

Supplementary_Material_bbag238

## Data Availability

Classification metrics from MTClass, as well as association *P*-values from MultiPhen and MANOVA, for all described studies are available via Zenodo (https://doi.org/10.5281/zenodo.10911584). The GTEx expression data used for this study can be found at https://gtexportal.org/, and the subset of data from the PsychENCODE CMC can be found via Synapse under accession syn12080241. The OneK1K single-cell gene expression data are publicly available via CELLxGENE.

## Code availability

The MTClass algorithm implements standardization of expression measures, model training, and classification using ensemble learning. The Python package is installable from PyPI (https://pypi.org/project/mtclass-ensemble/). The analysis code and example data are available on GitHub (https://github.com/ronnieli0114/MTClass).
