# Supplementary material for: A novel machine learning-based algorithm for eQTL identification reveals complex pleiotropic effects in the MHC region
Source: Brief Bioinform. 2026 May 19;27(3):bbag238. doi: 10.1093/bib/bbag238 (PMC13184528; doi:10.1093/bib/bbag238)
Supplement: Supplementary_Material_bbag238 [file supplementary_material_bbag238.zip › Supplementary_materials_BIB-25-0490_bbag238.R3_bbag238.docx]

Supplementary Materials and Figures for:

# A novel machine learning-based algorithm for eQTL identification reveals complex pleiotropic effects in the MHC region

Ronnie Y. Li^1^, Chang Su^2^, & Zhaohui S. Qin^2^

^1^ Neuroscience Graduate Program, Emory University, Atlanta, GA 30322

^2^ Department of Biostatistics and Bioinformatics, Emory University, Atlanta, GA 30322

## Supplementary Results

Simulation studies

We conducted a ranking challenge simulation to evaluate MTClass's ability to correctly prioritize true eQTL signals compared to MultiPhen and MANOVA under two scenarios representing linear and nonlinear genotype-phenotype relationships. The simulation tested 500 candidate variants, of which 10 were true regulatory signals, across a 5-dimensional expression matrix representing multi-tissue gene expression in 200 individuals.

In Scenario 1, where genotype classes differed by mean expression levels, all three methods demonstrated perfect signal recovery. MTClass, MultiPhen, and MANOVA each identified all 10 true signal SNPs in their top 10 ranked positions (100% recovery). The perfect lead SNP achieved an MCC of 0.66 in MTClass, while the 9 correlated LD variants achieved MCC values ranging from 0.48 to 0.68. MultiPhen and MANOVA reported highly significant p-values (p < 1 x 10^-9^) for all 10 signal SNPs. These results confirm that when genetic effects manifest as simple mean differences across tissues, MTClass performs equivalently to established linear methods **(Supplementary Figure 10A)**.

In Scenario 2, where genotype classes had identical means but different variances, the methods showed markedly different performance. MTClass successfully recovered 5 out of 10 true signal SNPs (50% recovery) in its top 10 ranked variants, including the perfect lead SNP which achieved an MCC of 0.34. The four additional recovered signals were among the LD variants most strongly correlated with the lead SNP, achieving MCC values ranging from 0.27 to 0.38. In stark contrast, MultiPhen and MANOVA completely failed to identify any of the 10 true signal SNPs in their top 10 ranked variants (0% recovery). The perfect lead SNP achieved p-values of 0.340 for MultiPhen and 0.370 for MANOVA **(Supplementary Figure 10B)**.

These simulation results reveal that when genotype-phenotype relationships conform to linear, additive models, all methods perform well. However, when regulatory effects manifest through variance modulation or other nonlinear mechanisms, MTClass maintains partial detection capability while linear methods completely lose power. The 50% recovery rate achieved by MTClass in the variance difference scenario demonstrates that ensemble classifiers can leverage information about expression variability to distinguish genotypes, even when average expression levels are identical. These results suggest that traditional multi-tissue eQTL mapping approaches may systematically miss variants whose regulatory effects involve modulation of expression stability or other non-additive mechanisms.

To assess whether MTClass can detect eQTL signals arising from different underlying genetic architectures, we evaluated classification performance under three genetic effect models (dominant, additive, and multiplicative) as well as a null model with no genotype-expression association, across three minor allele frequencies (MAF = 5%, 10%, and 20%). Under the null model, where all three genotype classes shared identical mean expression (μ = 1 per tissue), MTClass produced MCC values indistinguishable from zero across all MAF levels (mean MCC = −0.001, −0.002, and 0.010 at MAF = 5%, 10%, and 20%, respectively), confirming that the classifier does not generate spurious signal in the absence of a true genotype-expression association. Under all three effect models, MTClass achieved MCC values substantially above the null and well above the MCC = 0.30 threshold for meaningful classification signal across all MAF levels tested. Performance increased monotonically with MAF, reflecting the greater statistical power available when minor homozygotes are more common in the sample. Importantly, the additive and multiplicative models performed comparably to one another and only modestly below the dominant model at each MAF level, demonstrating that MTClass is not exclusively sensitive to the dominant genetic architecture that is perfectly aligned with its binary encoding. For example, at MAF = 20%, the dominant model achieved a mean MCC of 0.882 (SD = 0.034), the additive model achieved 0.713 (SD = 0.060), and the multiplicative model achieved 0.720 (SD = 0.054). These results demonstrate that MTClass can detect eQTL signals arising from a range of genetic effect models with reasonable power, even though its binary encoding is most naturally aligned with the dominant model (**Supplementary Figure 11**).

Examples from the 9-tissue case

We delved more deeply into the eGene-eQTL pairs that were classified very well by MTClass. One of the eQTL-eGene pairs with a high macro F1 score and MCC across all 3 iterations of the 9-tissue case was PPIE-chr1_39738050_T_C_b38 (rs12074147), which achieved a median macro F1 score of 0.981 and a median MCC of 0.962. This pair was also deemed highly significant by MultiPhen (p = 3.26 x 10^-23^) and MANOVA (p = 1.23 x 10^-21^). Indeed, dimensionality reduction using Uniform Manifold Approximation and Projection (UMAP) shows high separability between the two genotypes (**Supplementary Figure 12A**). According to the GTEx Consortium, this SNP is a significant eQTL in 49 out of 54 tissues (90.7%), including all 9 of the tissues in this study. Moreover, the PPIE gene is expressed highly in many tissues, especially testis, EBV-transformed lymphocytes, and the tibial nerve. PPIE is a cyclophilin, which means it participates in many important biological processes, including mitochondrial metabolism, apoptosis, and inflammation [1]. Diseases associated with PPIE include ischemic reperfusion injury, influenza, and certain cancers [2]. Biologically, given the plethora of functions served by PPIE, it is reasonable that this eQTL would be significant in many somatic tissues.

Next, we surveyed the eQTLs that were classified well by MTClass (median F1 > 0.60) but not by MultiPhen or MANOVA . An interesting example of such an eQTL is HLA-DQB1-chr6_32658276_A_T_b38 (rs28633132). For this eGene-eQTL pair, MTClass reported a median macro F1 score of 0.633 and median MCC of 0.290, while MultiPhen reported a p-value of 0.00145, and MANOVA reported a p-value of 0.101. UMAP dimensionality reduction analysis shows that the two genotype groups are not easily separable, suggesting a more complex relationship that would be better captured by MTClass (**Supplementary Figure 12B**). This gene plays a critical role in the immune system, as it is the human version of MHC class II. This SNP is a significant eQTL in 19 out of 54 GTEx tissues (35.2%), including 5 out of the 9 tissues (55.6%) that were tested in this case. Thus, it is conceivable that this eGene-eQTL pair would have far-reaching effects as a multi-tissue eQTL.

Examples from the brain tissue case

One eGene-eQTL pair that was identified in the brain tissue case was ERAP2-chr5_96916885_T_C_b38 (rs2910686), which achieved a median macro F1 of 0.936 and median MCC of 0.874 across 3 iterations. This pair was also reported as highly significant by MultiPhen (p = 7.32 x 10^-57^) and MANOVA (p = 8.95 x 10^-41^). UMAP dimensionality reduction analysis shows highly separable genotypes (**Supplementary Figure 12C**). According to the GTEx Consortium, this SNP is a significant eQTL in 49 out of 54 GTEx tissues (90.1%), including all 13 brain tissues tested. Although this gene’s expression profile is not specific to the brain, ERAP2 does play important roles in innate immunity and major histocompatibility complex class I (MHC Class I) antigen processing and presentation [3]. This suggests that ERAP2 might contribute to widespread immune processes that can ultimately impact the brain.

Next, we examined the eGene-eQTL pairs that were classified well by MTClass (median macro F1 score > 0.60) but showed weak associations according to MultiPhen and MANOVA . An interesting eQTL we discovered was RP11-867G23.10-chr11_66411592_T_C_b38 (rs12785651). MTClass reported a median macro F1 score of 0.682 and median MCC of 0.381, while MultiPhen reported a p-value of 0.00168 and MANOVA reported a p-value of 0.0114. The genotypes are not separable in UMAP space, but there are subtle differences in expression levels between the two genotypes that MTClass was still able to capture (**Supplementary Figure 12D**). RP11-867G23.10 is also known as NPAS4, and it is a transcription factor expressed in neurons that regulates the excitatory-inhibitory balance in the brain [4]. This eQTL is a significant eQTL in 3 out of 13 brain tissues (23.0%) according to the GTEx Consortium: cerebellum, cerebellar hemisphere, and cortex. As a transcriptional regulator, NPAS4 potentially has overarching effects on brain function, but the relationship between genotype and phenotype might be a more nonlinear one, as evidenced by MTClass’ differential ability to detect it.

Examples from the multi-tissue and multi-exon 2D study

One eGene-eQTL pair that achieved a perfect macro F1 score and MCC in the 2D study was FAM118A-chr22_45315973_G_A_b38 (rs104664). This SNP is in the intronic region of the FAM118A gene on chromosome 22. It is an eQTL in 49 out of 54 tissues (90.7%), including all 9 tissues used in this study. Intriguingly, this SNP is also an sQTL in 50 out of 54 tissues (92.6%), including all 9 tissues used in this study. When examining the normalized gene expression between genotypes, it is evident that the two genotypes have markedly different expression patterns across multiple exons and multiple tissues **(Supplementary Figure 13A)**. Upon nonlinear dimensionality reduction via UMAP, the genotypes are clearly highly separable **(Supplementary Figure 13B)**, providing a possible explanation for our method’s ability to classify this SNP well. This SNP has been reported as a significant eQTL in human osteoblasts [5], sputum [6], as well as CD4+ lymphocytes [7], suggesting that it has complex, cross-tissue effects on gene expression within the body. Furthermore, FAM118A is an integral membrane protein, and although its exact functions remain unclear, it is highly expressed in many regions of the body, suggesting it has important functions across several somatic tissues.

Furthermore, an eQTL that exhibits a more nonlinear relationship between genotype and multiple phenotypes is GTF2IP12-chr4_119444292_T_C_b38 (rs9759690). This eQTL achieved a perfect macro F1 score and MCC. According to the GTEx Portal, this is a significant eQTL in 36 out of 54 tissues (66.7%), including six out of the nine tissues (66.7%) used in this study. It is also a significant sQTL in 19 out of 54 tissues (35.2%), including six out of the nine tissues (66.7%) used in this study, demonstrating that it is indeed a multi-tissue, multi-exon eQTL. **Supplementary Figure 13C** shows that the relationship between genotype and phenotype is complex. Intriguingly, principal component analysis (PCA) was less able to separate the genotypes compared to UMAP, a nonlinear dimension reduction technique **(Supplementary Figure 13D)**. This suggests that the genotype-phenotype relationship is probably more nonlinear and therefore better captured by MTClass.

Examples from the PsychENCODE isoQTL study

We provide an anecdotal example of an isoGene-isoQTL pair (RGS7-1_241290879_T_G_b37 (rs10926417)) that was classified well by MTClass but did not achieve statistical significance in either MultiPhen or MANOVA. MTClass achieved a reasonably high median macro F1 score of 0.60 and a median MCC of 0.204, while MultiPhen reported a p-value of 0.153, and MANOVA reported a p-value of 0.172. Dimensionality reduction using t-SNE suggests that the two genotypes are tightly intertwined, with a few regions where one genotype predominates. However, examining the isoforms separately, it is evident that while the mean expression levels are similar across isoforms, the variance of the mutant genotype in isoforms 3 and 7 is significantly higher than that of the wild-type genotype **(Supplementary Figure 14A)**. This suggests a potential dysregulation of the expression of these specific RGS7 isoforms in individuals with the mutant genotype. Importantly, RGS7 is highly abundant in the brain, with the highest median expression in the cerebellar hemisphere and the frontal cortex **(Supplementary Figure 14B)**. As a regulator of G-protein signaling, RGS7 has been linked to intellectual disability and depression-related behaviors [8,9].

## Supplementary Methods

Intuition

MTClass employs a classification-based framework to identify multivariate eQTLs. The core principle is intuitive: if a genetic variant truly influences gene expression across multiple tissues or exons, then an individual's multi-source expression pattern should be predictive of their genotype at that variant. Better classification performance indicates a stronger, more consistent relationship between the variant and expression levels across contexts.

Classifier architecture

The input to each classifier is a feature vector of gene expression levels for a single donor. The specific features depend on the study design. In multi-tissue studies, the feature vector consists of expression levels of one gene across multiple tissues, such as 9, 13, or 48 tissue measurements per donor. In multi-exon studies, the features represent expression levels of multiple exons from one gene within a single tissue, typically 10 or more exon measurements per donor. For the 2D study, we used a flattened matrix containing expression levels across both tissues and exons for one gene, creating a high-dimensional feature vector. For each gene-variant pair tested, every donor in the dataset contributes one feature vector along with their corresponding genotype label.

All classifiers predict genotype classes at the variant being tested. We employed a dominant genetic model with two classes. Class 0 represents the homozygous reference genotype (A/A), while Class 1 represents carriers of at least one alternate allele (A/a or a/a). This binary classification scheme reduces complexity while capturing the question of whether carrying the variant alters multi-source expression patterns in a detectable way.

The Random Forest (RF) and Support Vector Machine (SVM) ensemble was used for all studies *except* the 2D exon-tissue study. For the Random Forest classifier, we used 150 trees with a maximum depth of 7, minimum samples split of 5, and minimum samples per leaf of 2. At each split, the number of features considered was set to the square root of the total number of features. Class probabilities were obtained through ensemble voting across all trees in the forest.

For the Support Vector Machine, we employed a Radial Basis Function (RBF) kernel with a regularization parameter (C) of 1.0 and a kernel coefficient (gamma) set to 'auto’. Since SVMs do not inherently produce probability estimates, we obtained class probabilities via Platt scaling, which fits a logistic regression model to the SVM's decision values.

Our ensemble strategy employed soft voting. For each donor, we averaged the class probabilities from the Random Forest and SVM, then assigned the genotype class with the highest average probability (argmax). This approach leverages the complementary strengths of both classifiers, with Random Forest providing robustness to noisy features and SVM capturing complex decision boundaries.

The Multi-Layer Perceptron (MLP) was used specifically for the 2D study involving tissue by exon expression data. The network architecture begins with an input layer that accepts the flattened feature vector with dimensions equal to the number of tissues multiplied by the number of exons. This is followed by a single hidden layer with half the initial number of neurons using the Rectified Linear Unit (ReLU) activation function. The output layer contains 2 neurons with softmax activation to produce class probabilities for the two genotype classes.

We trained the MLP using the Adam optimizer with a learning rate of 0.001, processing data in batches of 16 samples. Training proceeded for up to 30 epochs with early stopping implemented. The MLP was chosen for the 2D study because neural networks excel at learning hierarchical representations from high-dimensional data with complex interactions between features, making them particularly well-suited for capturing relationships between tissues and exons simultaneously.

For each gene-variant pair, we performed 4-fold cross-validation to assess classification performance. To ensure robustness and reproducibility, we repeated the entire cross-validation process 3 times using different random seeds. The median macro F1 score and Matthews Correlation Coefficient (MCC) across the 3 iterations were computed and used to rank variants.

We evaluated classifier performance using two complementary metrics. The macro F1 score represents the harmonic mean of precision and recall, averaged across both genotype classes:

Macro F1 = (F1_0_ + F1_1_) / 2

where F1ᵢ = 2 × (precision_i_ × recall_i_) / (precision_i_ + recall_i_) for genotype class i ∈ {0,1}. The macro F1 ranges from 0 to 1 where 1 indicates perfect classification. The Matthews Correlation Coefficient (MCC) is a balanced measure that accounts for class imbalance:

MCC = (TP × TN - FP × FN) / √ [(TP + FP)(TP + FN)(TN + FP)(TN + FN)]

where TP, TN, FP, and FN represent true positives, true negatives, false positives, and false negatives, respectively. MCC ranges from -1 to 1, where 1 indicates perfect classification, 0 indicates performance no better than random chance, and -1 indicates complete disagreement between predictions and true labels.

Higher classification scores indicate that the multi-source expression pattern is highly informative of the genotype, suggesting the variant has a strong, consistent regulatory effect across multiple contexts. Variants achieving near-perfect classification, with F1 scores and MCC values approaching 1.0, represent multi-phenotype eQTLs where expression levels across tissues or exons are tightly coupled to the genotype. Conversely, poor classification performance, with F1 scores near 0.5 and MCC values near 0, suggests no meaningful relationship, as the classifier cannot distinguish genotypes better than random guessing.

By ranking variants based on classification performance rather than p-values, MTClass identifies genetic variants with the strongest multi-source regulatory effects. These top-ranked variants are most likely to represent biologically relevant pleiotropic eQTLs that influence gene expression consistently across multiple tissues or regulatory contexts.

Nevertheless, we acknowledge that for downstream testing purposes such as Mendelian Randomization and Transcriptome-wide Association Studies, p-values and effect sizes are required. Thus, we devised a permutation-based approach to calculate an empirical effect size and p-value from the null distribution. To validate these permutation-derived statistics, we examined their correlation with classification metrics among significant eQTLs (empirical p < 0.05) from the 9-tissue study. We observed strong positive correlations between −log₁₀ p-value and both macro F1 score (Pearson R = 0.591, p < 0.001) and MCC (R = 0.598, p < 0.001), as well as between standardized effect size and macro F1 (R = 0.459, p < 0.001) and MCC (R = 0.497, p < 0.001) (**Supplementary Figure 15**). These correlations demonstrate that permutation-derived statistics appropriately reflect classification performance, supporting their validity for integrating MTClass results into established causal inference frameworks while maintaining our method's core advantage of detecting complex multi-tissue regulatory effects.

Simulation studies

To objectively evaluate MTClass's ability to identify true eQTL signals compared to existing linear methods under controlled conditions with known ground truth, we designed a ranking challenge simulation. We simulated a single gene with expression measured across 5 dimensions (representing different tissues) for 200 individuals evenly divided into two genotype classes (100 per class). We generated 500 candidate genetic variants to be tested for association with the multi-tissue expression pattern, of which only 10 were true signal SNPs with genuine regulatory effects, while the remaining 490 were null variants with no association to expression levels. The expression matrix remained constant across all variant tests, simulating the biological reality that gene expression is measured once per individual and then tested for association with many genetic variants.

The genotype matrix consisted of 500 SNPs × 200 individuals. The first SNP was the "perfect" lead SNP with genotypes perfectly aligned to the expression-generating model. To simulate realistic genetic architecture with linkage disequilibrium, we generated 9 additional signal SNPs highly correlated with the lead SNP by randomly flipping 5% of genotypes from the perfect vector (approximately 90% correlation). The remaining 490 SNPs were null variants generated by randomly permuting the genotype vector, maintaining the 100/100 class balance but destroying any correlation with expression patterns.

We evaluated two scenarios representing different genotype-phenotype relationships. In Scenario 1 (mean difference), the two genotype classes differed by mean expression levels while maintaining equal variances: genotype 0 from *N*(μ=0, σ²=1) and genotype 1 from *N*(μ=1, σ²=1). This represents a simple additive genetic effect readily detectable by linear methods. In Scenario 2 (variance difference), the genotype classes had identical means but different variances: genotype 0 from *N*(μ=0, σ²=1) and genotype 1 from *N*(μ=0, σ²=2). This creates a nonlinear relationship that violates linear method assumptions, as the genotype affects expression variability rather than central tendency.

We applied MTClass, MultiPhen, and MANOVA to all 500 variants. MTClass variants were ranked by descending MCC (calculated on out-of-fold predictions), while MultiPhen and MANOVA were ranked by ascending p-value. The primary evaluation metric was signal recovery in the top 10 ranked variants: how many of the 10 true signal SNPs each method successfully identified.

To evaluate MTClass's ability to detect signals under different genetic architectures, we simulated a cohort of 200 individuals with gene expression measured across 5 tissues, represented as a multivariate normal distribution with an identity covariance matrix (σ² = 1 per tissue). Genotypes for a single SNP were sampled under Hardy-Weinberg equilibrium at three minor allele frequencies (MAF = 5%, 10%, and 20%). Four genetic models were considered, each defining the per-tissue expression mean vector (μ) for each of the three genotype classes (AA, Aa, aa): a null model with μ_AA_ = μ_Aa_ = μ_aa_ = 1 (no effect); a dominant model with μ_AA_ = 0 and μ_Aa_= μ_aa_ = 1.5; an additive model with μ_AA_ = 0, μ_Aa_ = 1, and μ_aa_ = 2; and a multiplicative model with μ_AA_ = 1, μ_Aa_ = 2, and μ_aa_ = 4. In all cases, the mean vector was identical across all five tissues within a genotype class. A fixed dominant encoding was applied throughout, assigning AA individuals to one class and {Aa, aa} individuals to the other, consistent with MTClass's default binary encoding. Classification performance was measured by the MCC averaged across folds. Each condition was repeated across 100 independent replicates, and results are reported as mean MCC ± standard deviation across replicates.

Imputation of gene expression measures

To impute missing expression measures for the brain tissue case (13 tissues) and the 48-tissue case (48 tissues), we tested a variety of multiple imputation techniques. Multiple imputation iteratively treats each feature as the response variable in a linear regression, using a combination of the known values of other features to predict the missing values [10,11].

We randomly masked 50% of the 927 samples for the 9-tissue case to test imputation strategies. The pattern of missing data was similar to the real GTEx data in the brain tissue and 48-tissue cases. For each gene, we measured the Pearson correlation coefficient (R^2^) between the predicted values and the actual values. We tested five multiple imputation techniques: (1) support vector regression [12], (2) Bayesian ridge regression [13], (3) random forest regression [14], (4) predictive mean matching [15], and (5) K-nearest neighbors regression [16]. Overall, we found that predictive mean matching yielded the highest median R^2^, so we imputed the expression levels for the brain tissue case using this method **(Supplementary Figure 16)**.

Comparison with other methods

MultiPhen [17] performs a “reverse” ordinal regression on the genotypes to test for the significance of association with multiple phenotypes. Here, the variant genotypes become the dependent variable, and the phenotypes become the predictor variables. Proportional odds logistic regression is used to define the probabilities for each class (allele count):

$$P\left( X_{ig}\leq m \right)=\frac{1}{1+\exp\left( -\alpha_{gm}-\sum_{1}^{K} \beta_{gk}Y_{ik} \right)}$$

We used the mPhen() function in the MultiPhen R package with default parameters to run the analysis. MultiPhen is installable from CRAN (<http://cran.r-project.org/>) or any CRAN mirror, with documentation available at <http://cran.r-project.org/web/packages/MultiPhen/MultiPhen.pdf>.

Multivariate analysis of variance (MANOVA) [18] is a statistical test commonly used to test the significance of association between a predictor variable (genotype) and several response variables (phenotypes). MANOVA assumes that the phenotypes are normally distributed and that there is no multicollinearity between the dependent variables. We used the built-in manova() function in the R language with default parameters to conduct the analysis. The dependent variables were the K phenotypes, and the independent variables were the variant genotypes. By default, the manova() function in R tests the significance of the Pillai trace statistic.

Canonical correlation analysis (CCA) identifies and measures the associations among two sets of variables, and it is especially useful when there are multiple intercorrelated variables such as phenotypes. CCA has been implemented in mv-PLINK [19], but because we did not have access to the software, we implemented a modified version using the cancor() function in R. We then tested the statistical significance of the correlation value using the Wilks’ lambda statistic in the CCP package in R.

Akin to MultiPhen, SCOPA and META-SCOPA [20] utilize a reverse linear regression in which the genotype is the outcome variable and the phenotypes are the predictor variables. However, due to the unavailability of the software, we implemented a modified version of the model but used logistic regression to more effectively represent the binarized genotypes. Specifically, we compared the full model, in which all phenotypes were used as predictors, to a null model in which no predictors were used. We used analysis of variance to calculate a p-value quantifying the improvement in model fit achieved by adding the predictors.

Finally, we calculated nominal p-values for each eGene-eQTL pair in each of the 13 individual brain tissues using tensorQTL [21]. We used all covariates computed by the GTEx Consortium version 8 in the analysis. Next, we combined the 13 p-values using the Cauchy combination test [22] to obtain a single p-value for each eGene-eQTL pair. The Cauchy combination test was implemented in the ACAT package in R.

Variant annotation

For each of the MTClass results, the top eQTLs by MCC from the top 10 eGenes were annotated using Ensembl’s Variant Effect Predictor (VEP, release 113) [23]: <https://useast.ensembl.org/info/docs/tools/vep/index.html> . The GRCh37 version of the VEP was used for the isoQTL and OneK1K scRNA-seq results, while the GRCh38.p14 assembly was used for all others. To resolve duplicate annotations, only the most severe functional consequence and one rsID was kept for each variant.

### References

1. Yao Q, Li M, Yang H, et al. Roles of Cyclophilins in Cancers and Other Organ Systems. World Journal of Surgery 2005; 29:276–280

2. Kim JO, Nau MM, Allikian KA, et al. Co-amplification of a novel cyclophilin-like gene (PPIE) with L-myc in small cell lung cancer cell lines. Oncogene 1998; 17:1019–26

3. de Castro JAL, Stratikos E. Intracellular antigen processing by ERAP2: Molecular mechanism and roles in health and disease. Hum Immunol 2019; 80:310–317

4. Pollina EA, Gilliam DT, Landau AT, et al. A NPAS4-NuA4 complex couples synaptic activity to DNA repair. Nature 2023; 614:732–741

5. Kwan T, Grundberg E, Koka V, et al. Tissue Effect on Genetic Control of Transcript Isoform Variation. PLoS Genetics 2009; 5:e1000608

6. Qiu W, Cho MH, Riley JH, et al. Genetics of Sputum Gene Expression in Chronic Obstructive Pulmonary Disease. PLoS one 2011; 6:e24395

7. Murphy A, Chu J-H, Xu M, et al. Mapping of numerous disease-associated expression polymorphisms in primary peripheral blood CD4+ lymphocytes. Human Molecular Genetics 2010; 19:4745–4757

8. Hill WD, Davies G, Liewald DC, et al. Examining non-syndromic autosomal recessive intellectual disability (NS-ARID) genes for an enriched association with intelligence differences. Intelligence 2016; 54:80–89

9. Sutton LP, Khalatyan N, Savas JN, et al. Striatal RGS7 Regulates Depression-Related Behaviors and Stress-Induced Reinstatement of Cocaine Conditioned Place Preference. eNeuro 2021; 8:

10. White IR, Royston P, Wood AM. Multiple imputation using chained equations: Issues and guidance for practice. Stat Med 2011; 30:377–99

11. Buuren SV, Groothuis-Oudshoorn K. mice: Multivariate Imputation by Chained Equations in R. J. Stat. Soft. 2011; 45:

12. Wang X, Li A, Jiang Z, et al. Missing value estimation for DNA microarray gene expression data by Support Vector Regression imputation and orthogonal coding scheme. BMC Bioinformatics 2006; 7:32

13. Xu W, Liu X, Leng F, et al. Blood-based multi-tissue gene expression inference with Bayesian ridge regression. Bioinformatics 2020; 36:3788–3794

14. Hong S, Lynn HS. Accuracy of random-forest-based imputation of missing data in the presence of non-normality, non-linearity, and interaction. BMC Medical Research Methodology 2020; 20:199

15. E. F. Akmam, T. Siswantining, S. M. Soemartojo, et al. Multiple Imputation with Predictive Mean Matching Method for Numerical Missing Data. 2019; 1–6

16. Pan R, Yang T, Cao J, et al. Missing data imputation by K nearest neighbours based on grey relational structure and mutual information. Applied Intelligence 2015; 43:614–632

17. O’Reilly PF, Hoggart CJ, Pomyen Y, et al. MultiPhen: Joint Model of Multiple Phenotypes Can Increase Discovery in GWAS. PLoS one 2012; 7:e34861

18. Yang Q, Wang Y. Methods for Analyzing Multivariate Phenotypes in Genetic Association Studies. Journal of Probability and Statistics 2012; 2012:1–13

19. Ferreira MA, Purcell SM. A multivariate test of association. Bioinformatics 2009; 25:132–3

20. Mägi R, Suleimanov YV, Clarke GM, et al. SCOPA and META-SCOPA: software for the analysis and aggregation of genome-wide association studies of multiple correlated phenotypes. BMC Bioinformatics 2017; 18:25

21. Taylor-Weiner A, Aguet F, Haradhvala NJ, et al. Scaling computational genomics to millions of individuals with GPUs. Genome Biol 2019; 20:228

22. Liu Y, Xie J. Cauchy combination test: a powerful test with analytic p-value calculation under arbitrary dependency structures. J Am Stat Assoc 2020; 115:393–402

23. McLaren W, Gil L, Hunt SE, et al. The Ensembl Variant Effect Predictor. Genome Biol 2016; 17:122

### Supplementary Figures


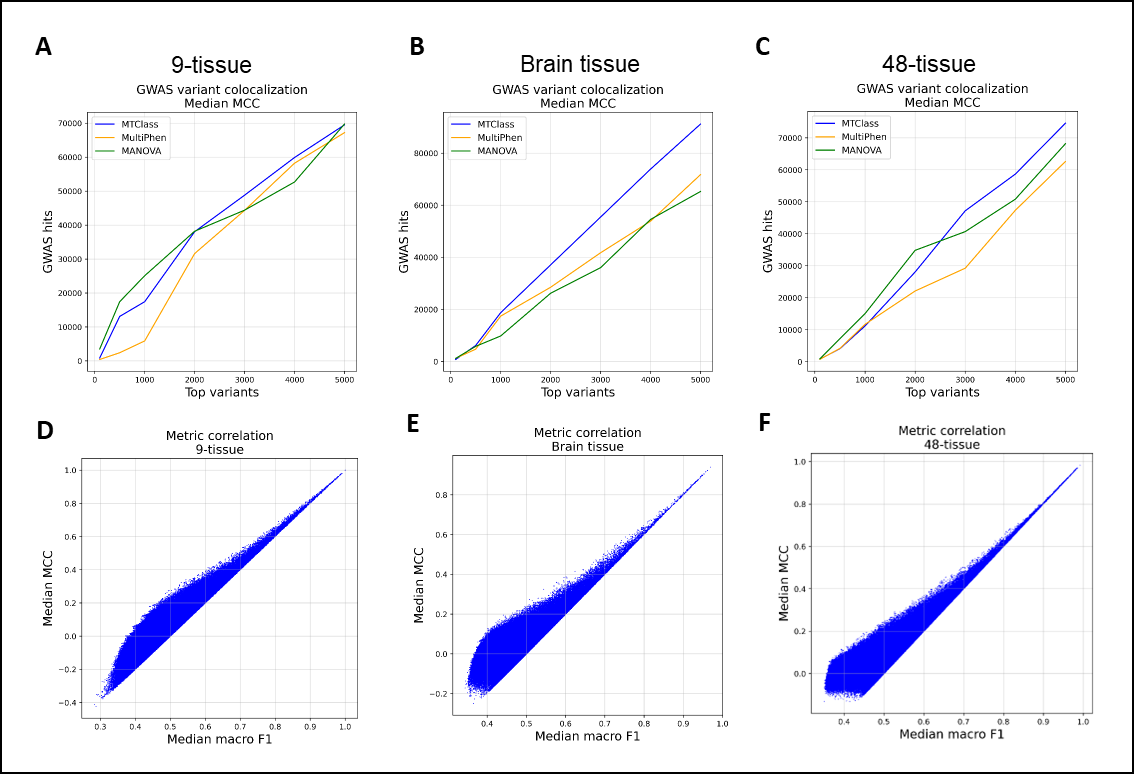


**Supplementary Figure 1.** **A-C.** GWAS variant colocalization analysis using the median Matthews correlation coefficient (MCC) across 3 iterations produces similar results as using median macro F1 score in **A.** the 9-tissue case, **B.** the brain tissue case, and **C.** the 48-tissue case. **D-F.** Correlation between the median macro F1 score and median MCC score in **D.** the 9-tissue case, **E.** the brain tissue case, and **F.** the 48-tissue case. In all cases, the top variants showed particularly high correlation between these two metrics.


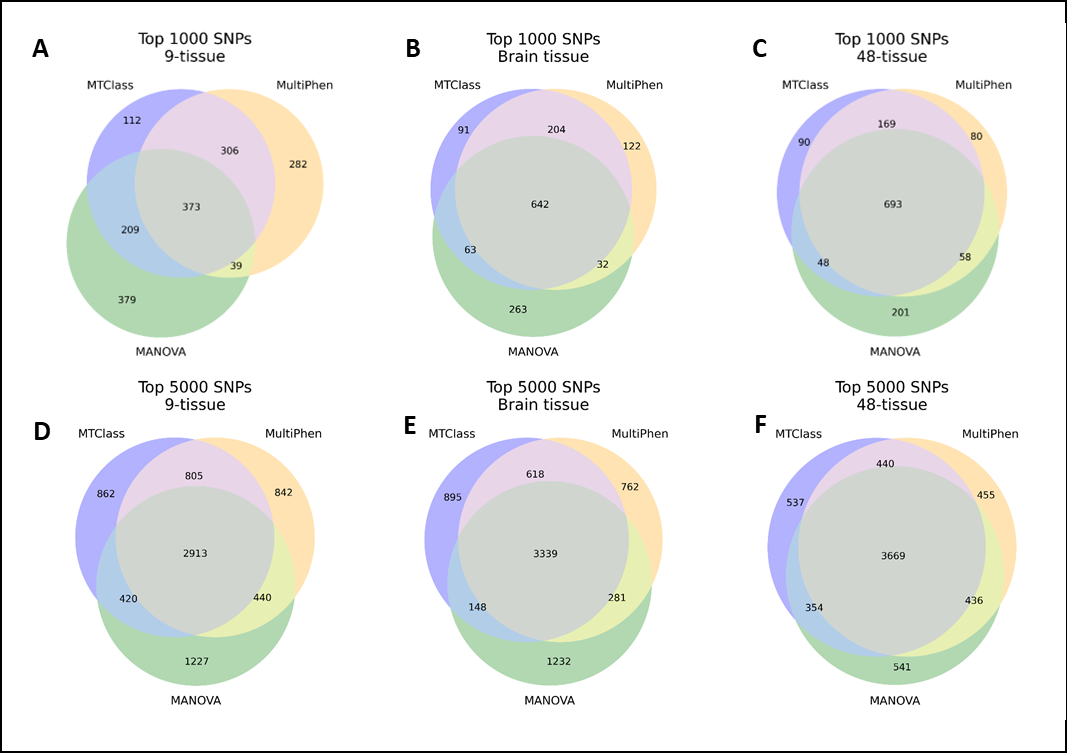


**Supplementary Figure 2.** **A-C.** Overlap among the top 1000 variants for the **A.** 9-tissue case, **B.** brain tissue case, and **C.** 48-tissue case. **D-F.** Overlap among the top 5,000 SNPs for the **D.** 9-tissue case, **E.** brain tissue case, and **F.** 48-tissue case. Overall, there is a significant amount of overlap in the top variants detected among the three methods. However, each method still identifies a substantial number of unique variants.


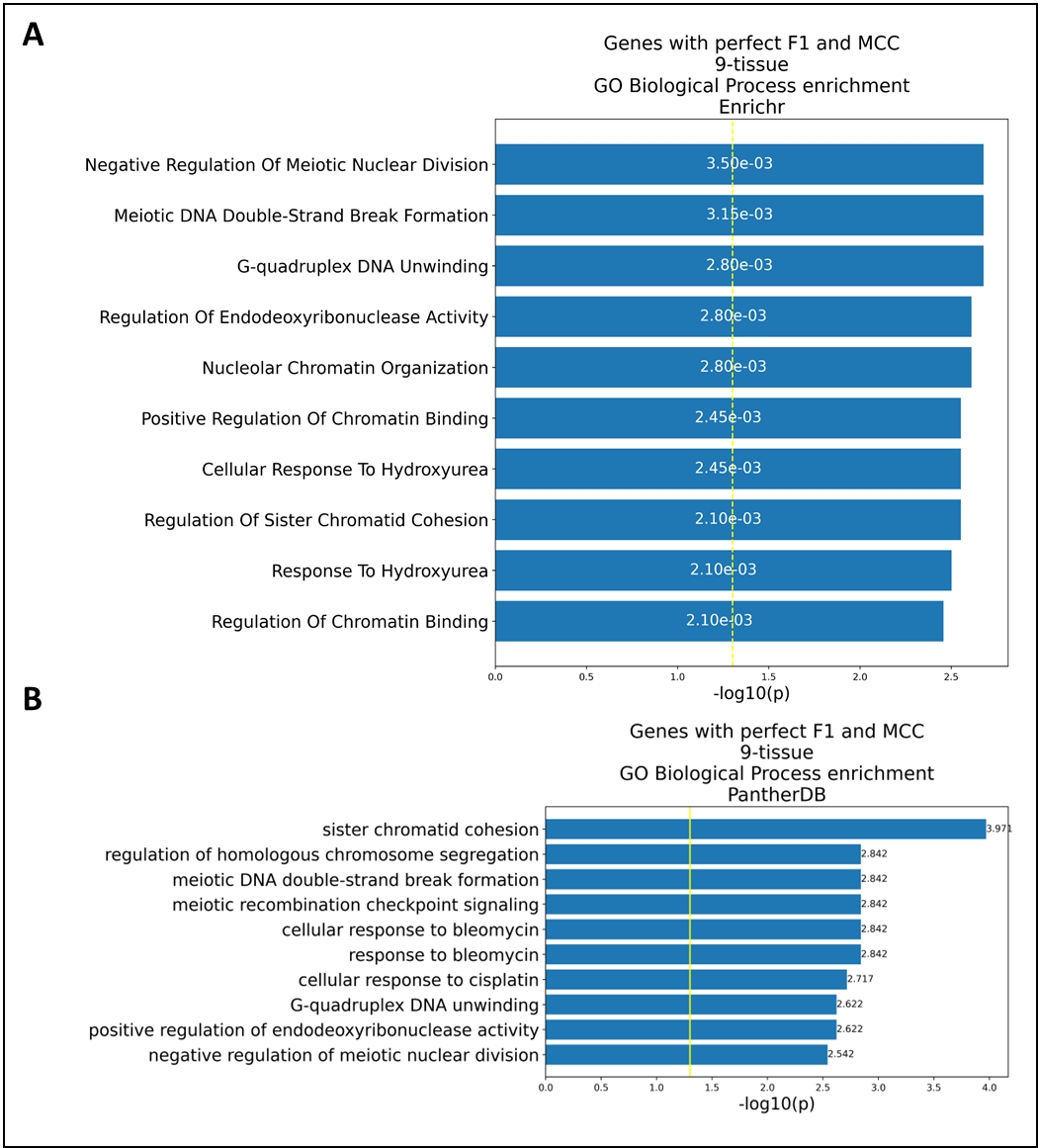


**Supplementary Figure 3.** Gene set enrichment analysis of the 8 genes in the 9-tissue case that contained variants with perfect classification performance. These genes are enriched for nuclear and cell division processes. **A.** Enrichment analysis of the 8 genes in the 9-tissue case using Enrichr. **B.** Enrichment analysis of the same genes using PantherDB.


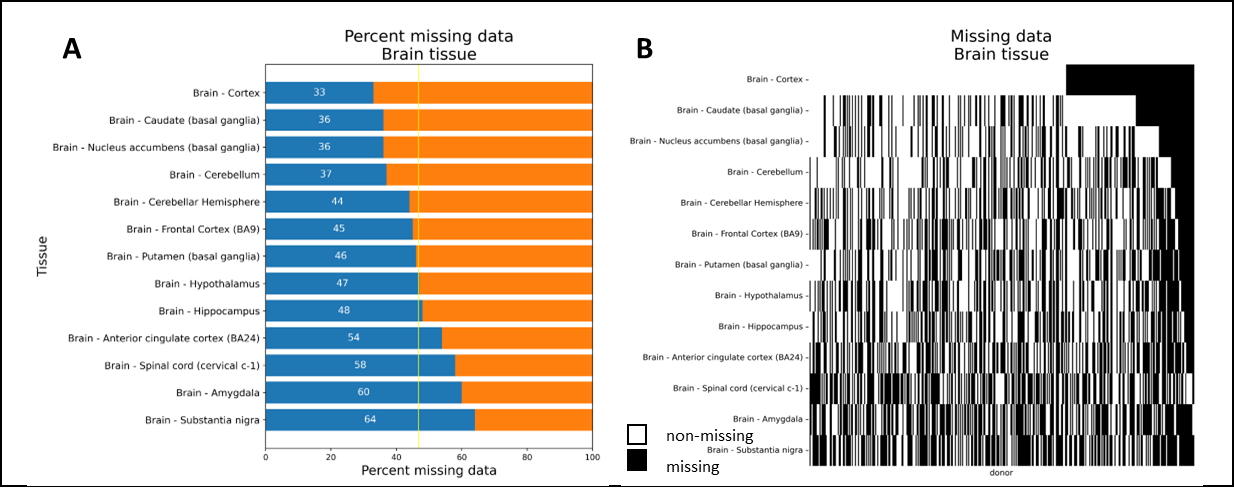


**Supplementary Figure 4.** Visualization of missing data in the multi-tissue, brain tissue case. The brain tissue case was missing approximately 47% of the data on average. **A.** Percent missing data by tissue. **B.** Heatmap of missing donor-tissue combinations.


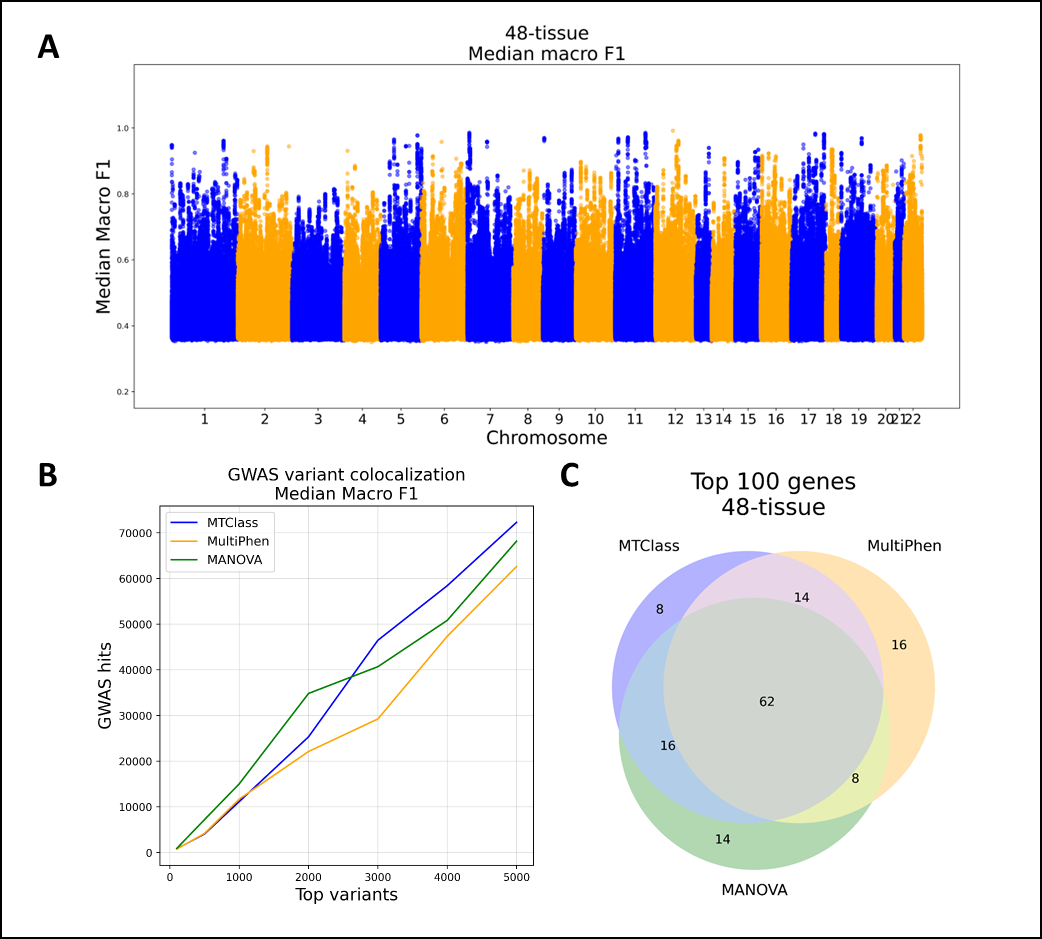


**Supplementary Figure 5.** Results from the 48-tissue case (multi-tissue study). **A**. Manhattan plot of variants according to their median macro F1 score. **B.** GWAS variant colocalization analysis of top variants from each method. **C.** Overlap of top 100 genes detected by each method.


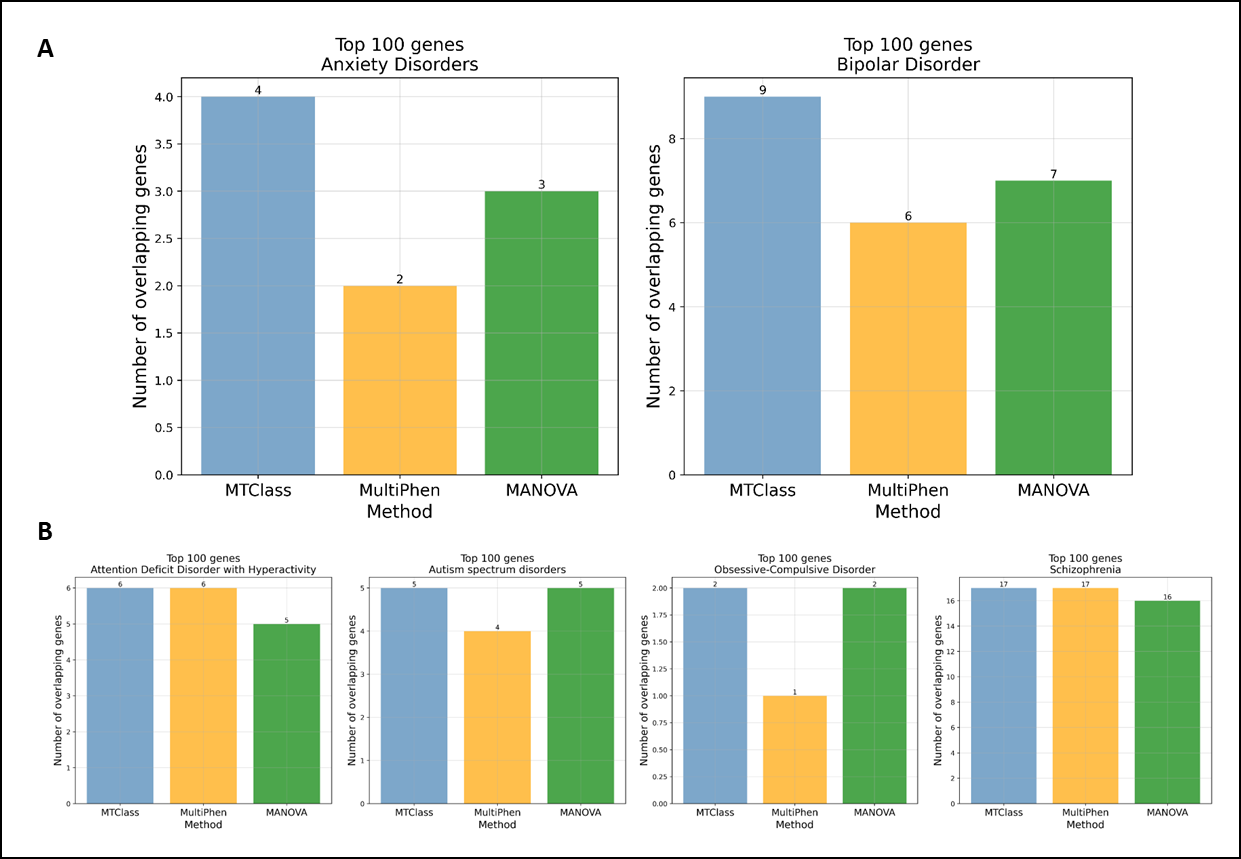


**Supplementary Figure 6.** Disease gene overlap analysis in the PsychENCODE isoQTL study with prefrontal cortex-related traits. **A.** MTClass detects more eGenes that overlap with anxiety disorders and bipolar disorder than MultiPhen and MANOVA. **B.** For other disorders such as attention deficit-hyperactivity disorder (ADHD), autism spectrum disorder (ASD), obsessive-compulsive disorder (OCD), and schizophrenia, although MTClass does not always singly detect the greatest number of disease-associated genes, our method always ties with the leading method in the analysis.


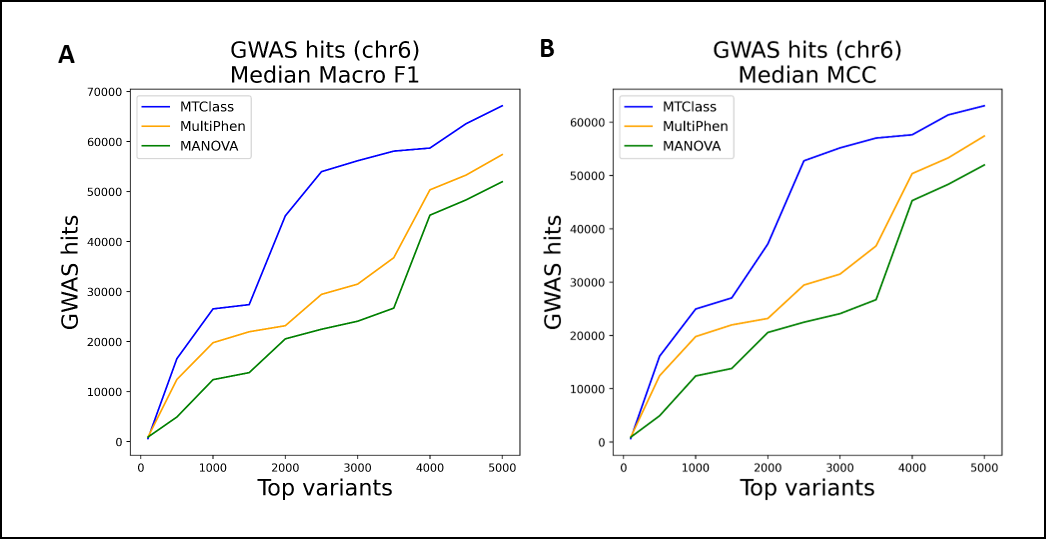


**Supplementary Figure 7.** GWAS variant colocalization analysis on the OneK1K scRNA-seq cohort shows that hits on chr6 contribute to the majority of the total GWAS hits among the top variants, after sorting by both **A.** median macro F1 and **B.** median MCC. Due to the abundance of HLA genes on chr6, we concluded that immune-related features were of great importance, especially to the MTClass algorithm.


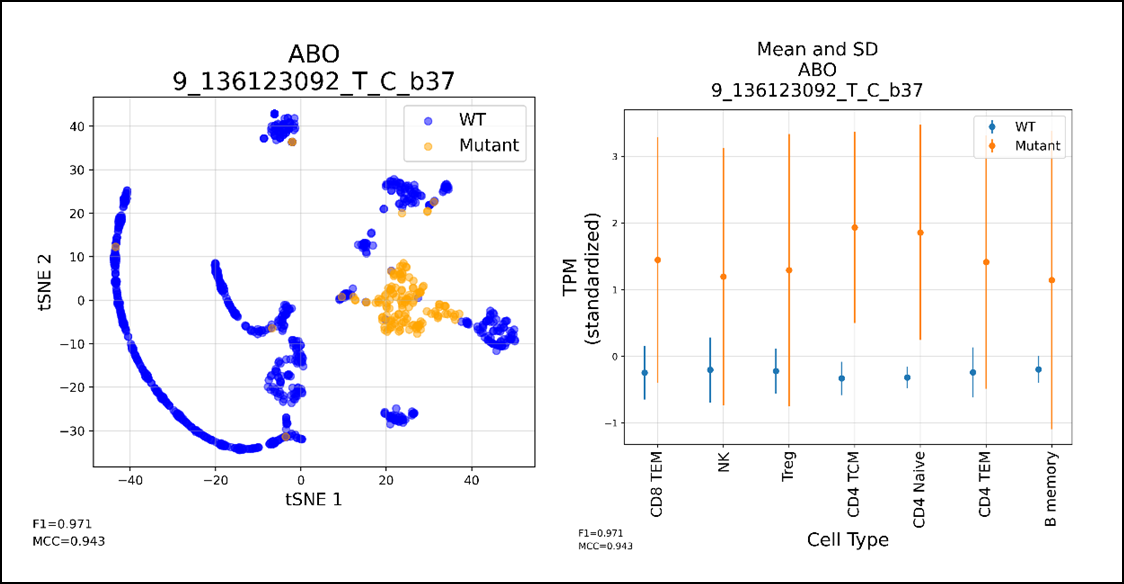


**Supplementary Figure 8.** Anecdotal example of eQTLs from the OneK1K scRNA-seq study. The top MTClass result is a SNP in the ABO gene, which helps to determine human blood typing. UMAP analysis shows highly separable genotypes (left). The mutant genotype has both higher expression and higher variance in this gene compared to the wild type genotype in all seven cell types (right).


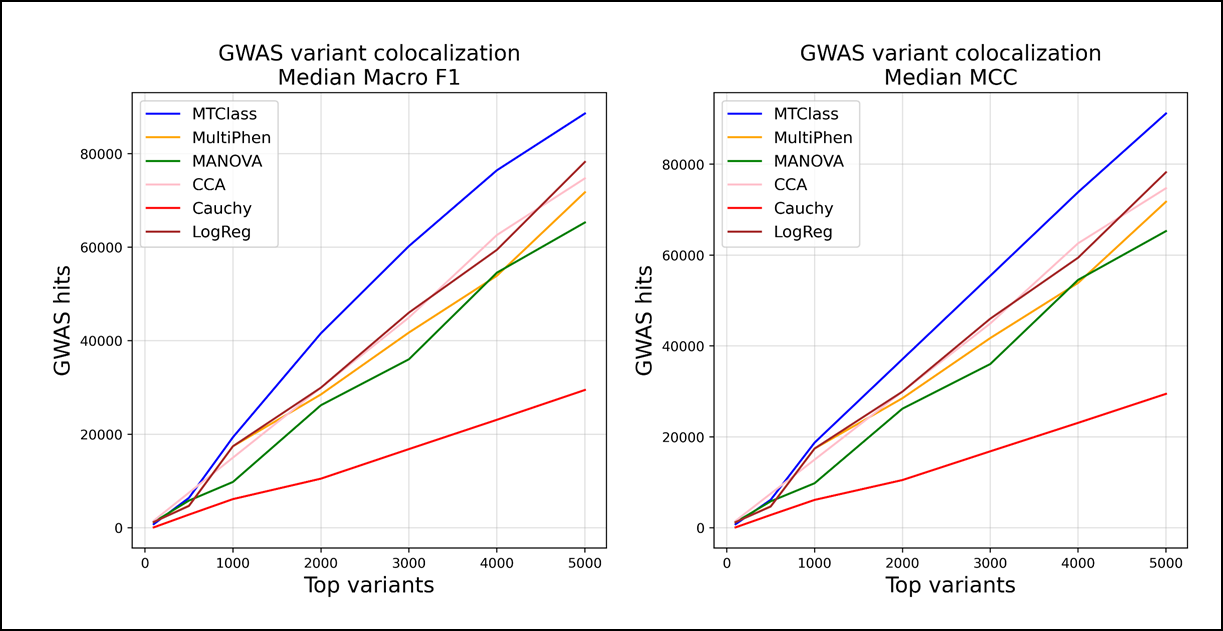


**Supplementary Figure 9.** Extended performance comparison with more multivariate association methods in the brain tissue case shows that MTClass is superior at detecting eQTLs of high functional importance. In addition to MultiPhen and MANOVA, we compared MTClass to canonical correlation analysis (CCA), reverse logistic regression, and the Cauchy combination test.


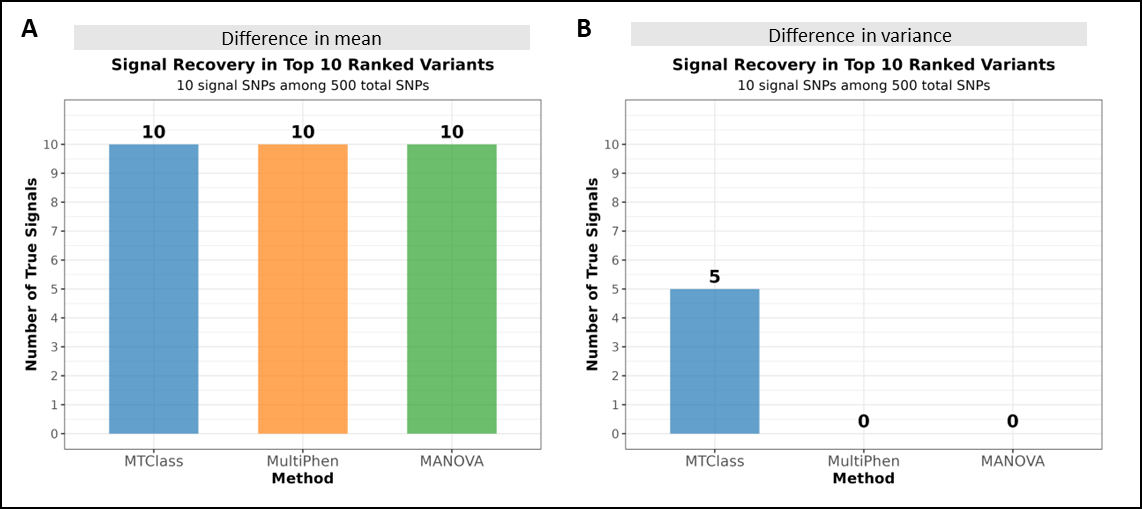


**Supplementary Figure 10.** Simulation study utilizing nonlinear genotype-phenotype relationships to demonstrate the utility of MTClass. **A.** Expression matrix contained only a difference in mean of the normal distribution between the two genotype classes. Bars show the number of top eQTLs recovered (out of 10) after ranking the results. **B.** Expression matrix contained a difference in variance (no difference in mean) of the normal distribution between the two genotype classes. Bars show the number of top eQTLs recovered.


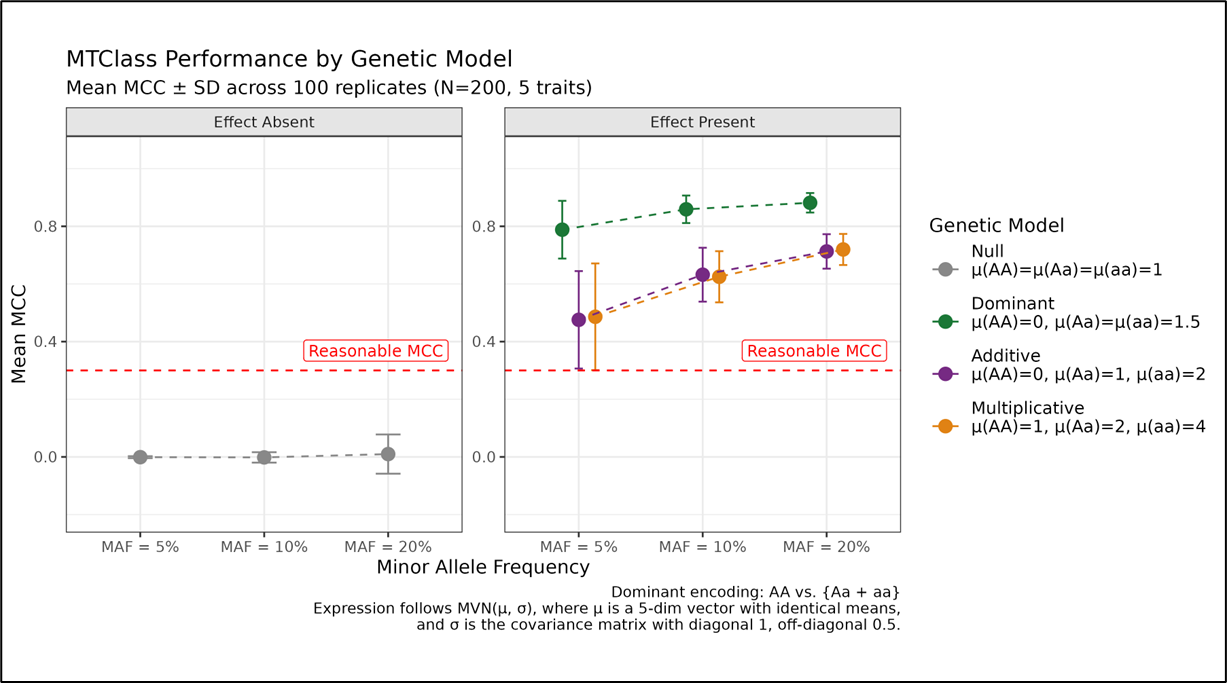


**Supplementary Figure 11.** MTClass retains the ability to detect additive and multiplicative genetic effects with high MCC values, despite its default binary genotype encoding. When there is no effect across genotypes, MTClass performs at chance level (MCC = 0, left panel). However, when there is a detectable difference across genotypes, MTClass performs well regardless of the underlying assumed genetic model, shown by the high MCC scores. A reasonable MCC was defined to be 0.30 (dashed red line) based on previous literature and fraction of phenotypic variance explained. MCC values are presented as means $\pm$ standard deviation across 100 replicates.


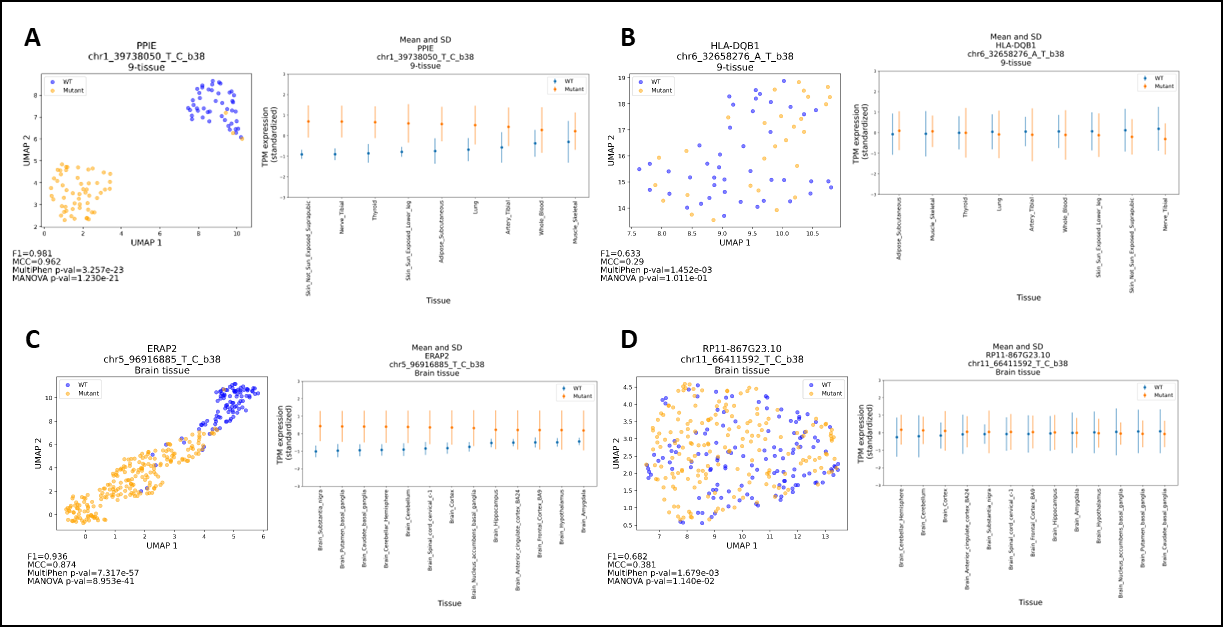


**Supplementary Figure 12. A-B.** Anecdotal examples of significant eQTL-eGene pairs in the 9-tissue case. **A.** PPIE-chr1_39738050_T_C_b38 (rs12074147) was reported as highly significant by all three methods. UMAP analysis of this pair (left) and the expression levels across 9 tissues (right). **B.** HLA-DQB1-chr6_32658276_A_T_b38 (rs28633132) was classified well by MTClass but not reported as significant by MultiPhen and MANOVA. **C-D.** Anecdotal examples of significant eQTL-eGene pairs in the brain tissue case. **C.** ERAP2-chr5_96916885_T_C_b38 (rs2910686) was reported as highly significant by MultiPhen and MANOVA. UMAP analysis of this pair (left) and the expression levels across 13 brain tissues (right). **D.** RP11-867G23.10-chr11_66411592_T_C_b38 (rs12785651) was classified well by MTClass but not reported as significant by MultiPhen and MANOVA.


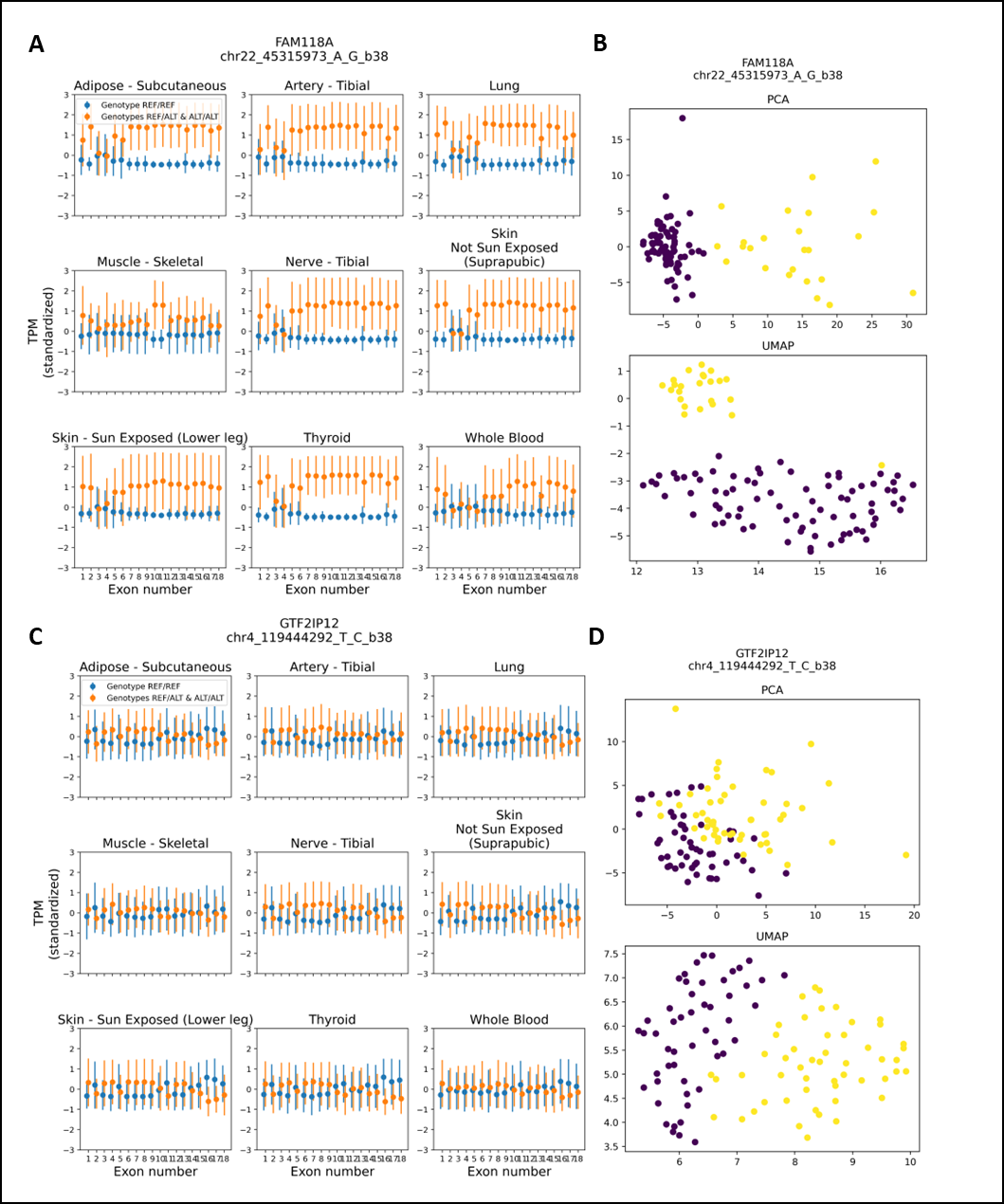


**Supplementary Figure 13.** Anecdotal examples of eGene-eQTL pairs classified well by MTClass across multiple exons and tissues in the 2D study. **A.** Mean and standard deviation plots of standardized expression levels in TPM across all exons and 9 tissues. This eGene-eQTL pair shows differences in gene expression in multiple exons and multiple tissues. **B.** Both PCA and UMAP dimensionality reduction show high separability between the two genotypes. **C.** Expression levels in TPM across exons and tissues for an eGene-eQTL pair exhibiting less separability between genotypes. **D.** Linear dimensionality reduction with PCA cannot separate the genotypes as neatly as nonlinear techniques such as UMAP, suggesting the relationship is more nonlinear.


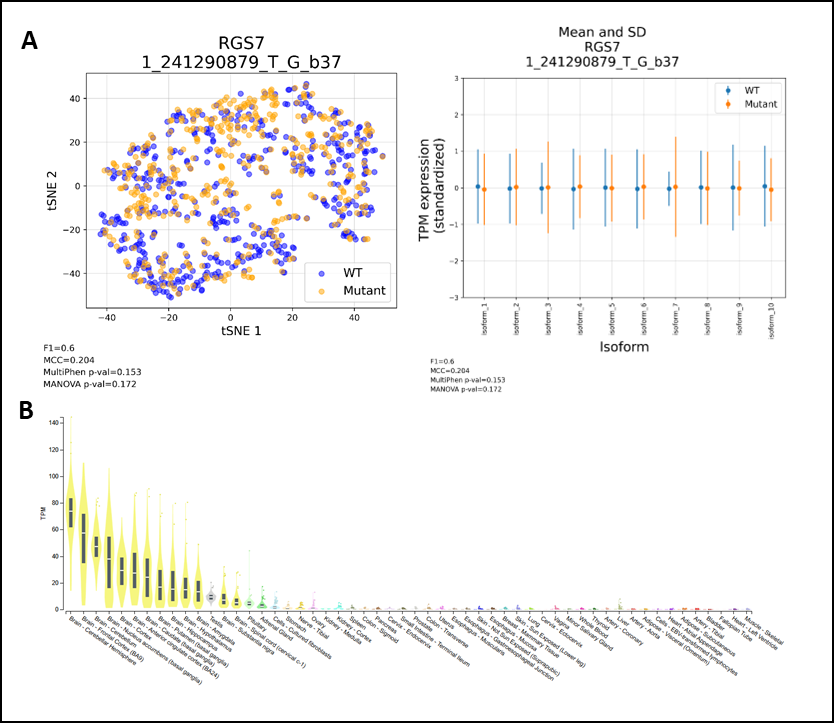


**Supplementary Figure 14.** RGS7-1_241290879_T_G_b37 (rs10926417) is an anecdotal example of an isoGene-isoQTL pair detected by MTClass but not by other methods. **A.** t-SNE dimensionality reduction (left) of this eQTL shows poor separation of genotypes, but suggestive regions where one genotype predominates. This isoGene shows expression differences across multiple isoforms and differences in variance in isoforms 3 and 7, where the mutant genotype has higher variation compared to the wild type genotype (right). **C.** According to the GTEx Consortium, the RGS7 gene is most highly expressed in the brain, especially in the frontal cortex.


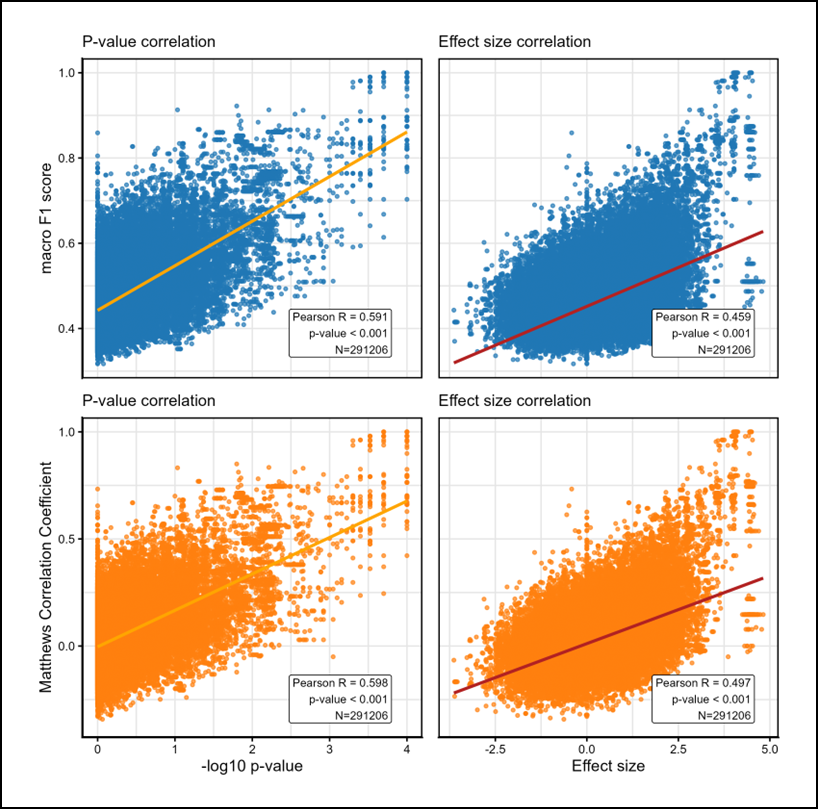


**Supplementary Figure 15.** Correlation between classification metrics and permutation-derived statistics for significant eQTLs in the 9-tissue study. Scatterplots showing the relationship between MTClass classification performance metrics (macro F1 score, top row; Matthews Correlation Coefficient, bottom row) and permutation-derived statistics (−log_10_ p-value, left column; standardized effect size, right column) for 291,206 gene-variant pairs with empirical p < 0.05 in the 9-tissue case. Each point represents a single eQTL. Regression lines (orange for macro F1, red for MCC) indicate positive correlations. Pearson correlation coefficients and p-values are displayed in each panel. Strong positive correlations demonstrate that permutation-derived p-values and effect sizes appropriately reflect classification performance, validating their use for downstream analyses.


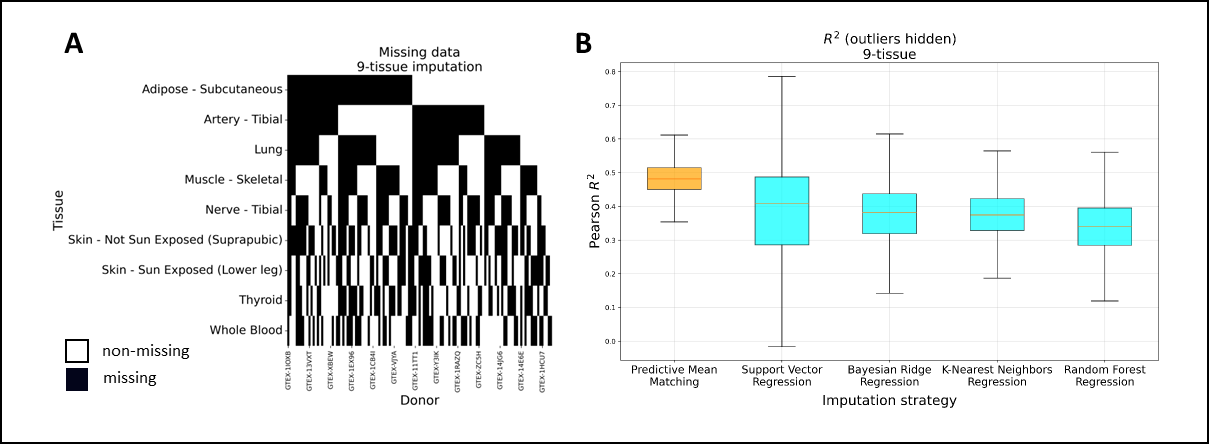


**Supplementary Figure 16.** Missing data in the 9-tissue imputation study. 50% of the donor-tissue combinations were randomly removed from the 9-tissue case, where the ground truth is known, to test various imputation strategies. **A.** Heatmap of randomly removed donor-tissue combinations. **B.** Boxplot of Pearson correlation coefficient between true and imputed gene expression measures by imputation strategy. Predictive mean matching had the highest median $R^{2}$**.**
